# Dynamic three-dimensional structures of a metal–organic framework captured with femtosecond serial crystallography

**DOI:** 10.1038/s41557-024-01460-w

**Published:** 2024-03-25

**Authors:** Jaedong Kang, Yunbeom Lee, Seonggon Lee, Hosung Ki, Jungmin Kim, Jain Gu, Yongjun Cha, Jun Heo, Kyung Won Lee, Seong Ok Kim, Jaehyun Park, Sang-Youn Park, Sangsoo Kim, Rory Ma, Intae Eom, Minseok Kim, Jeongho Kim, Jae Hyuk Lee, Hyotcherl Ihee

**Affiliations:** 1 Department of Chemistry and KI for the BioCentury, Korea Advanced Institute of Science and Technology (KAIST), Daejeon, Republic of Korea; 2 Center for Advanced Reaction Dynamics, Institute for Basic Science (IBS), Daejeon, Republic of Korea; 3 Pohang Accelerator Laboratory, POSTECH, Pohang, Republic of Korea; 4 Department of Chemistry, Inha University, Incheon, Republic of Korea

**Keywords:** Reaction kinetics and dynamics, X-ray diffraction, Metal-organic frameworks

## Abstract

Crystalline systems consisting of small-molecule building blocks have emerged as promising materials with diverse applications. It is of great importance to characterize not only their static structures but also the conversion of their structures in response to external stimuli. Femtosecond time-resolved crystallography has the potential to probe the real-time dynamics of structural transitions, but, thus far, this has not been realized for chemical reactions in non-biological crystals. In this study, we applied time-resolved serial femtosecond crystallography (TR-SFX), a powerful technique for visualizing protein structural dynamics, to a metal–organic framework, consisting of Fe porphyrins and hexazirconium nodes, and elucidated its structural dynamics. The time-resolved electron density maps derived from the TR-SFX data unveil trifurcating structural pathways: coherent oscillatory movements of Zr and Fe atoms, a transient structure with the Fe porphyrins and Zr_6_ nodes undergoing doming and disordering movements, respectively, and a vibrationally hot structure with isotropic structural disorder. These findings demonstrate the feasibility of using TR-SFX to study chemical systems.

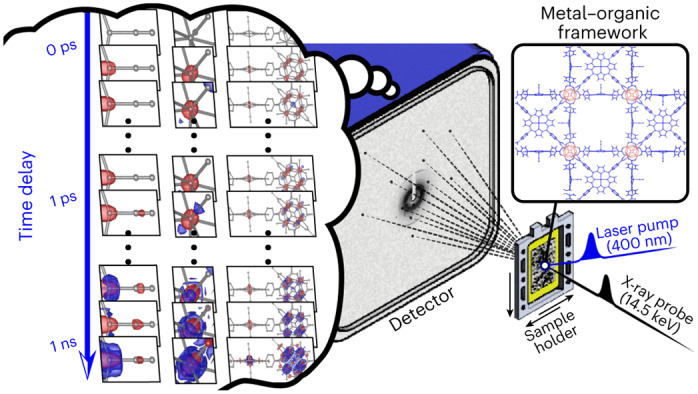

## Abstract (web summary)

Time-resolved femtosecond crystallography (TR-SFX) is a powerful technique to monitor structural transitions in protein crystals at the atomic level, but its use in non-protein synthetic materials remains limited. Now TR-SFX has been used to visualize the structural dynamics of metal–organic frameworks, showing the potential of this tool to study the dynamic motion of crystalline porous materials.

## Main

Novel crystalline materials consisting of organic, inorganic or organometallic building blocks have inspired intense investigation of their structures and chemical properties, as well as their applications as materials for the capture and separation of gases^1–5^. It is of great importance to characterize not only the static structures of those materials grown in single crystals but also their conversion in response to external stimuli^6,7^. Thus far, such structural characterization has been performed by comparing their static structures before and after the application of the stimuli. For example, photocrystallography at low temperatures is routinely conducted to capture the key intermediates of organic and inorganic reactions^8,9^. However, it would be desirable to probe the structural dynamics of crystalline small-molecule materials with a structural probe equipped with high-temporal resolution. In fact, femtosecond X-ray and electron diffraction techniques based on the detection of a few Bragg spots can provide some information on structural dynamics^10–12^, but whole electron density (ED) maps that visualize atomic positions in three dimensions cannot be constructed from such a limited number of Bragg spots. In other words, nearly all of the Bragg spots need to be collected to obtain an ED map via crystallography. It has been demonstrated that time-resolved crystallography at synchrotron radiation sources with a time resolution of ~100 ps can be applied to chemical systems^13^, but the method has limitations when studying crystalline samples undergoing irreversible or slow-recovery reactions and reactions that occur faster than 100 ps.

Time-resolved serial femtosecond crystallography (TR-SFX) is a promising tool for overcoming such limitations in studies of ultrafast structural dynamics. TR-SFX is an SFX technique^14^ that combines a pump-and-probe set-up that produces femtosecond pulses and thus provides both atomic-scale spatial and femtosecond time resolutions. With the development of X-ray free-electron lasers (XFELs), TR-SFX has emerged as a powerful tool for probing the fast dynamics of structural transitions in proteins^15–23^. In contrast, crystalline samples of small molecules, either in the form of molecular crystals or porous covalent frameworks, have never been studied with TR-SFX, although one study with static SFX has recently been reported^24^. In principle, TR-SFX should be applicable to any molecular system that can be prepared as micro-sized single crystals, but a number of technical obstacles have prevented its application to single crystals consisting of small molecules. For example, single crystals of small molecules tend to have a substantially smaller lattice constant than protein crystals. As a result, a diffraction pattern from a single crystal of small molecules exhibits much sparser Bragg peaks than a protein crystal, making the indexing of its diffraction spots difficult. In addition, because a small-molecule crystal generally has a higher absorption cross-section than a protein crystal, the optical laser pulse cannot penetrate the crystal effectively. Consequently, a small-molecule crystal generates a much weaker photoinduced signal than a protein crystal, making the data analysis more complicated.

**Fig. 1:**
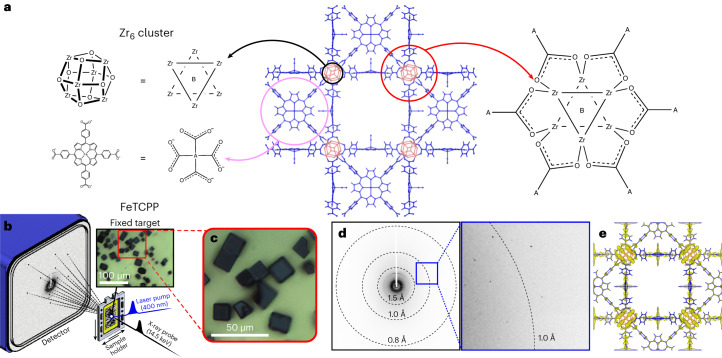
Structure of PCN–224(Fe)–CO and the TR-SFX set-up. **a**, Schematics of the molecular structure of PCN–224(Fe)–CO investigated in this work. PCN–224(Fe)–CO is composed of iron tetrakis(4-carboxylatophenyl)porphyrin (FeTCPP) molecules and Zr_6_ nodes (left). For clarity, CO ligands have been omitted from the FeTCPP representation. Each Zr_6_ node is connected to six FeTCPPs via the carboxylato group of FeTCPP to form the MOF (right). The CO ligated FeTCPPs and Zr_6_ nodes are indicated in blue and red, respectively. **b**, Schematic of the TR-SFX set-up with a fixed-target sample holder on which the micro-sized crystals are placed. **c**, A close-up view of the fixed target. Micro-sized crystals are visible. **d**, A typical TR-SFX pattern (left) and a magnified view of the selected region (right). The circular dashed lines show the resolution rings on the measured diffraction pattern. **e**, ED map of PCN–224(Fe)–CO at a negative time delay of −3.9 ps, obtained by TR-SFX in this study.

In this work, we successfully used TR-SFX to study a non-protein, metal–organic framework (MOF) system. Although the light-initiated dynamics of various MOFs have been investigated by various time-resolved spectroscopic techniques^25,26^, time-resolved crystallographic investigation of MOFs has not yet been reported. We prepared an iron–porphyrinic zirconium MOF^27^, denoted porous coordination network–224(Fe) (PCN–224(Fe)), in the form of micro-sized single crystals optimized for TR-SFX analysis. PCN–224(Fe) is a porphyrinic MOF in which the Fe porphyrin building blocks, which can act as photoantennae, are connected through hexazirconium (Zr_6_) nodes, as shown in Fig. 1a. One carbonyl (CO) ligand was further ligated to each of the Fe-porphyrin sites, resulting in PCN–224(Fe)–CO, which can undergo photoinduced CO dissociation. TR-SFX data for PCN–224(Fe)–CO were acquired at various time delays between the optical laser and X-ray probe pulses. As the unit cell of PCN–224(Fe) is smaller than that of a typical protein crystal, only sparse Bragg peaks were detected for an X-ray energy of 5–12 keV, the range typically used for the TR-SFX analysis of proteins. Therefore, we used an X-ray energy as high as 14.5 keV to increase the number of peaks in the detection area sufficiently to allow structure refinement.

## Result and discussion

### Experimental set-up and data collection

The TR-SFX set-up is shown in Fig. 1b. A fixed-target sample holder was employed instead of an injector-style crystal delivery system, such as a liquid-jet injector producing a stream of microcrystals dispersed in a liquid or gel, as generally used for TR-SFX experiments on protein crystals. The fixed-target sample holder has two major advantages over conventional injector-style crystal delivery systems. First, the amount of sample consumed in the experiment can be greatly reduced^28,29^. Second, there are no problems of chemical compatibility of the sample crystals with the medium because the crystals are simply placed on a substrate, such as a thin film, rather than dispersed in a liquid or gel-type medium. In our experiment, PCN–224(Fe)–CO crystals were placed on a sample holder consisting of a solid film within a frame, which was moved during measurement to allow a fresh single crystal to be exposed to the pump and probe pulses. The typical size of the crystals used in the experiment was 10–20 μm (Fig. 1c). The photoinduced ligand dissociation reaction within a crystal was triggered by a 400 nm femtosecond laser pulse, and after a time delay, a femtosecond X-ray pulse from an XFEL was directed towards the crystal to obtain a diffraction pattern. Data were collected across 33 time delays, significantly exceeding the number of time delays in previous TR-SFX studies, to quantitatively unveil the ultrafast dynamic motions within the crystal structure. A typical diffraction pattern is shown in Fig. 1d. Bragg peaks are sparse, but can be observed even at wide scattering angles beyond the spatial resolution of 1.0 Å. By indexing the diffraction patterns and extracting the scattering amplitudes, we obtained ED maps for all time delays. The ED maps at negative time delays (−3.9, −2.4, −0.6 and −0.2 ps), one of which is shown in Fig. 1e, reproduce the reported structure of PCN–224(Fe)–CO well, indicating that the SFX data are of high quality. We note that, due to the crystallographic disorder of the Zr atoms, the EDs of the Zr atoms in the Zr_6_ nodes show highly elongated features along a direction defined as the *d* axis^30^. The structure refinement of this ED map yielded the structure model shown in Fig. 1a. Crystallographic parameters and collection data are presented in Supplementary Tables 1–5.

### Time-resolved difference ED maps

**Fig. 2:**
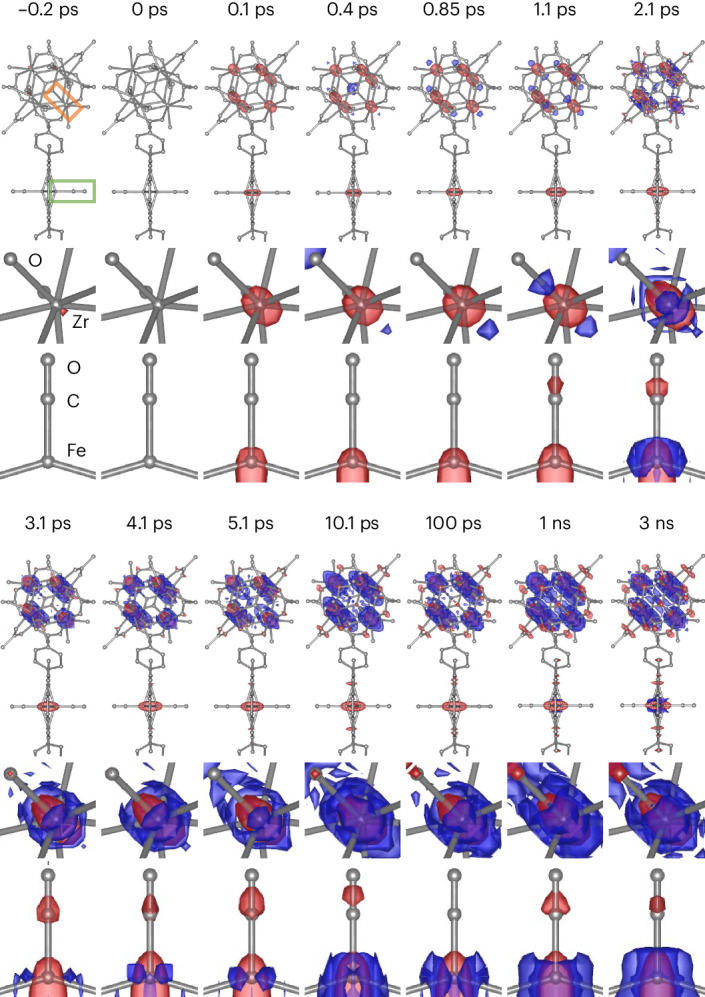
Time-resolved DED maps of PCN–224(Fe)–CO at selected time delays. The first and fourth rows show DED maps that include both the Fe porphyrin and Zr_6_ node contoured at −5.0*σ* (red, −0.58 e Å^−^^3^) and +5.0*σ* (blue, 0.58 e Å^−^^3^). The time delays are indicated above the maps. The second and fifth rows show DED maps of one of the Zr atoms in the Zr_6_ node (indicated by the orange box in the first map for −0.2 ps) contoured at −5.0*σ* (red, −0.58 e Å^−^^3^) and +5.0*σ* (blue, 0.58 e Å^−^^3^). The third and sixth rows show DED maps of the Fe porphyrin region contoured at −3.0*σ* (red, −0.35 e Å^−^^3^) and +3.0*σ* (blue, 0.35 e Å^−^^3^). The FeCO site is indicated by the green box in the first map for −0.2 ps after rotation by 90° for better visualization. Here, *σ* is defined as the average of root-mean-square (rms) values, where the rms value is calculated for each time delay by taking the rms of the DED values in the corresponding DED map.

**Extended Data Fig. 1:**
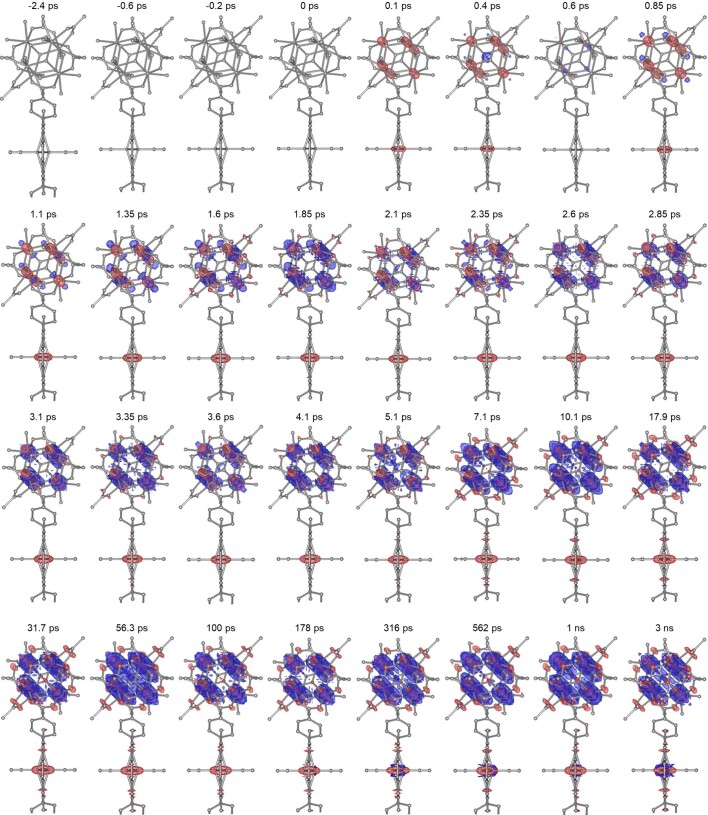
Time-resolved DED maps of PCN–224(Fe) at all time delays (high contour levels). DED maps including both the Fe-porphyrin and Zr_6_ node regions are shown. The red and blue colors denote the negative and positive electron densities, contoured at ±5.0σ (0.58 e Å^−3^), respectively. Here, the definition of the *σ* value is the same as the definition of the *σ* value in Fig. 2.

**Extended Data Fig. 2:**
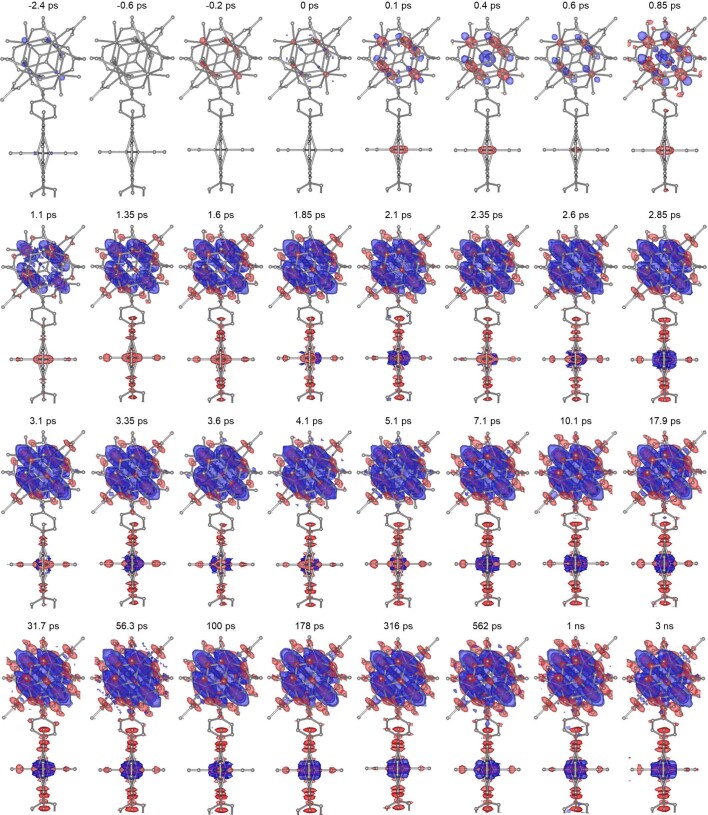
Time-resolved DED maps of PCN–224(Fe) at all time delays (low contour levels). DED maps including both the Fe-porphyrin and Zr_6_ node regions are shown. The red and blue colors denote the negative and positive electron densities, contoured at ±3.0σ (0.35 e Å^−3^), respectively. Here, the definition of the *σ* value is the same as the definition of the *σ* value in Fig. 2.

**Extended Data Fig. 3:**
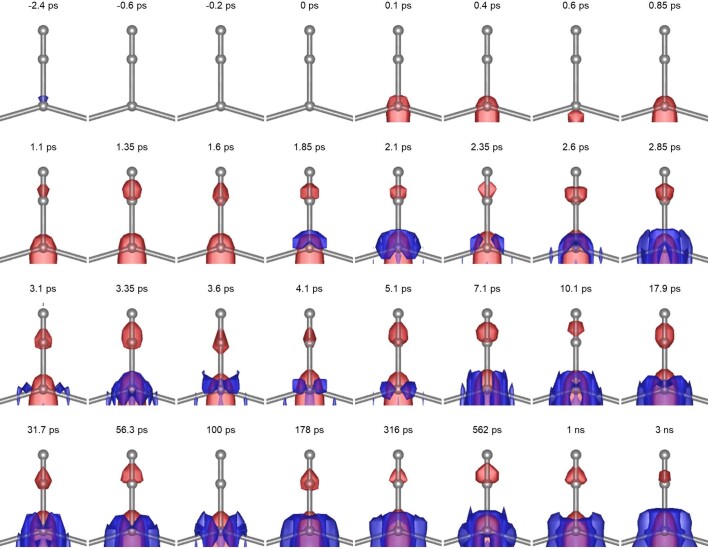
Time-resolved DED maps of PCN–224(Fe) at all time delays (magnified views for FeCO site). DED maps including Fe atom and the CO ligand in the Fe-porphyrin regions are shown. The red and blue colors denote the negative and positive electron densities, contoured at ±3.0σ (0.35 e Å^−3^), respectively. Here, the definition of the *σ* value is the same as the definition of the *σ* value in Fig. 2.

**Extended Data Fig. 4:**
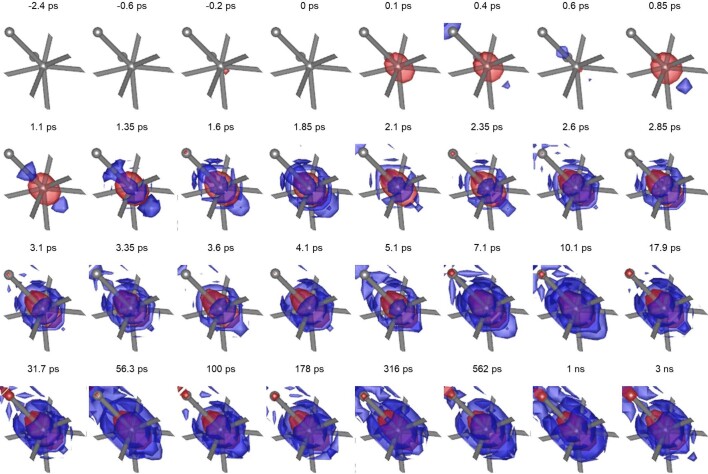
Time-resolved DED maps of PCN–224(Fe) at all time delays (magnified views for Zr_6_ node). DED maps including one Zr atom in Zr_6_ node regions are shown. The red and blue colors denote the negative and positive electron densities, contoured at ±5.0σ (0.58 e Å^−3^), respectively. Here, the definition of the *σ* value is the same as the definition of the *σ* value in Fig. 2.

**Extended Data Fig. 5:**
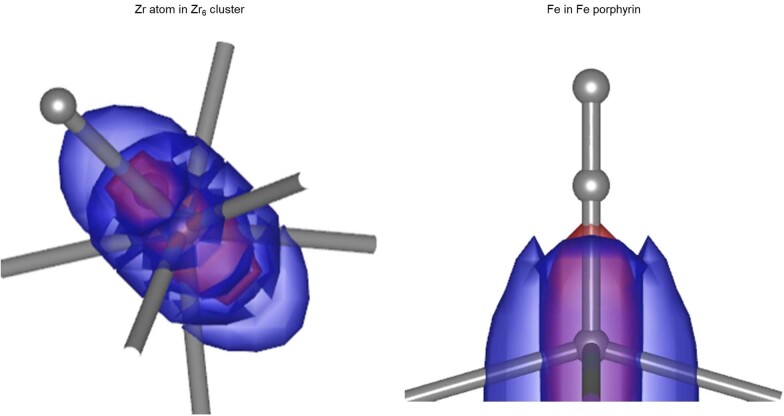
Simulated DED maps around Zr and Fe atoms for the vibrationally hot structure. The simulated DED maps around Zr atom (left) and Fe atom (right) obtained by subtracting the electron density map before the thermal excitation from that after the thermal excitation are shown. The red and blue colors denote the negative and positive electron densities, contoured at ±2σ (0.14 e Å^−3^ for Zr atom and 0.16 e Å^−3^ for Fe atom), respectively. The *σ* values for the maps around Zr and Fe represent the root-mean-square (rms) values of the simulated DED maps around Zr and Fe, respectively.

The difference structure factor amplitudes (Δ|*F*|) for each time delay were obtained by subtracting the structure factor amplitudes (|*F*|) for the pre-excited state, derived as the average from negative time delays of −3.9, −2.4, −0.6 and −0.2 ps, from the structure factor amplitudes at each respective time delay. Applying the difference Fourier approximation, the difference ED (DED; Δ*ρ*) maps at each time delay were produced by Fourier transformation of Δ|*F*|. The time-resolved DED maps of the whole unit cell shown in Fig. 2 (for selected time delays) and Extended Data Fig. 1 (for all time delays) indicate that strong DED signals are concentrated in the Zr_6_ nodes, with weaker DED signals observed in other regions. When the contours are scaled down, the Fe porphyrin region also shows distinct DED features (Extended Data Fig. 2). Close-up views of the Zr_6_ node and Fe porphyrin are shown in Fig. 2 (for selected time delays) and Extended Data Figs. 3 and 4 (for all time delays, Extended Data Fig. 3 for Fe porphyrin and Extended Data Fig. 4 for the Zr_6_ node). The DED map at 0.1 ps has a strong negative density at the Zr atom accompanied by two strong positive densities around the Zr atom along the *d* axis, indicating that the Zr atom is further disordered along this axis. Furthermore, at 0.1 ps, both the CO ligand and Fe atom show a negative density, indicating CO dissociation and displacement of Fe from the original position, respectively. These features become more evident at 1.1 ps as the intensities of the DED maps increase with time. The initially anisotropic features around the Zr and Fe atoms become less anisotropic over time. For example, at 10.1 ps, the negative densities around the original positions of the Zr and Fe atoms are surrounded more isotropically by positive density. Such features become saturated after tens of picoseconds and persist up to 3 ns, the longest time delay of our measurement. The DED features at the long time delays are in accord with the simulated DED map accounting for thermal excitation (Extended Data Fig. 5), implying the presence of vibrationally hot metal atoms. A closer inspection of the DED maps also reveals that all of the DED features exhibit periodic strengthening and weakening in the early time delays, hinting at coherent oscillation.

### Singular value decomposition and extraction of time constants

**Extended Data Fig. 6:**
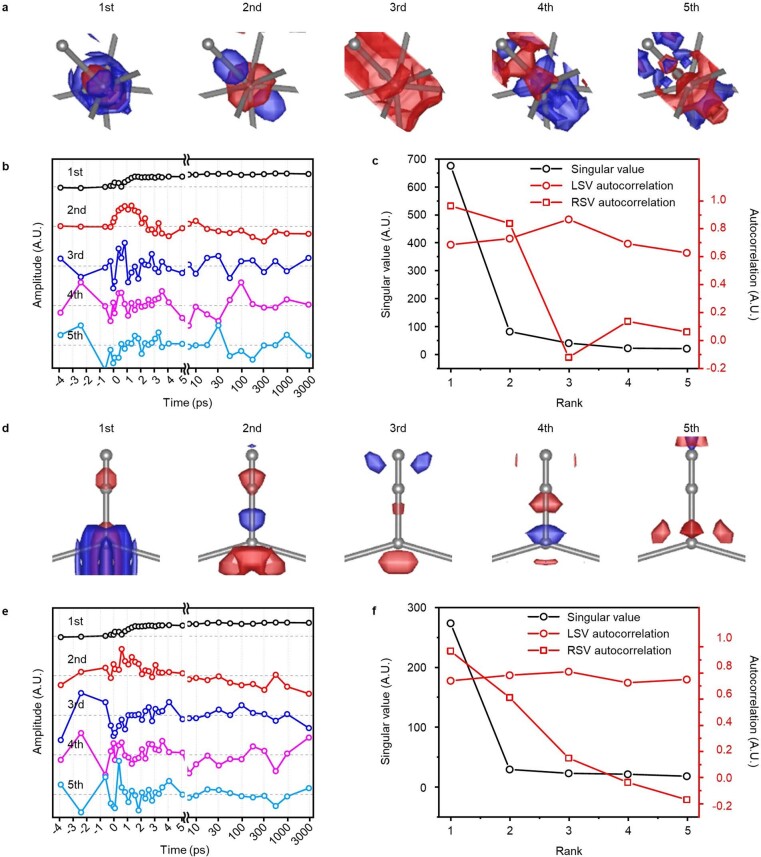
Results of singular value decomposition (SVD) and kinetic analyses on the DED maps of the Zr6 node and FeCO site. **a–c**, First five (**a**) LSVs, (**b**) RSVs, and (**c**) singular values obtained from the SVD analysis on the DED maps of the Zr_6_ node. In **c**, the autocorrelation values of the LSVs and RSVs are shown as well. **d–f**, The counterparts of **a–c** obtained from the SVD analysis on the DED maps of the FeCO site. In **a**, the red and blue colors denote the negative and positive electron densities, respectively. The 1st LSV is contoured at ±0.8σ (0.0043 e Å^−3^) and the 2nd to 5th LSVs are at ±1.0σ (0.0053, 0.0023, 0.0052 and 0.0053 e Å^−3^, respectively). In **d**, those colors indicate the negative and positive electron densities, respectively. the 1st LSV is contoured at ±1.0σ (0.0061 e Å^−3^) and the 2nd to 5th LSVs are countered at ±2.0σ (0.0104, 0.0122, 0.0122 and 0.112 e Å^−3^, respectively). σ of each LSV was calculated as the standard deviation of the difference electron density of the LSV. Each *σ* represents the rms value for each LSV. Source data

**Extended Data Fig. 7:**
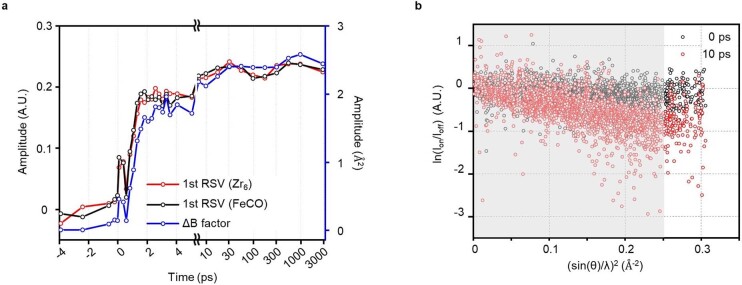
Comparison of the first right singular vectors (RSVs) obtained from the SVD analysis on the DED maps of the Zr6 node and FeCO site and time-resolved Wilson B-factors. **a**, Comparison of the two 1st RSVs of the DED maps of the Zr_6_ node (black) and FeCO site (red). Time-resolved Wilson B-factors (blue) are also shown. The two 1st RSVs of two metal sites coincide with each other. They are in accordance with the time trace of the Wilson B-factor, implying that thermal motions play a dominant role in this primary kinetic component. **b**, Here, I_off_ and I_on_ represent the diffraction intensities before and after photoexcitation, respectively. θ denotes half of the diffraction angle, and λ indicates the wavelength of the X-ray. The Wilson plots for 0 ps (black) and 10 ps (red) data. The data in the region from 0 to 0.25 Å^−2^ marked with gray were used to obtain ΔB.

To analyse the DED maps quantitatively, we used singular value decomposition (SVD)^31,32^ to decompose the original data into time-invariant DED features (left singular vectors (LSVs)), their relative contributions (singular values) and their time profiles (right singular vectors (RSVs)). Here, to focus on each of the changes near the Zr_6_ node and FeCO site, the two DED maps corresponding to these two regions were analysed separately by SVD analysis. Extended Data Fig. 6 shows the first LSVs and their RSVs for these two regions. For the DED maps around the Zr_6_ node and FeCO site, the first LSVs simply reflect the features that are dominant at late time delays, which were assigned to a vibrationally hot structure. Meanwhile, the first RSVs for the two regions have almost identical shapes (Extended Data Fig. 7), indicating that they represent the kinetics of thermal excitation of the entire structure of PCN–224(Fe)–CO or PCN–224(Fe) following photoexcitation. In addition, the time-resolved trace of the isotropic temperature factors (*B*-factors) is consistent with the first RSVs (Extended Data Fig. 7), further confirming that the first RSVs represent thermal kinetics. Inspection of the lower-rank RSVs revealed an additional major component (the second RSVs for the DED maps around the Zr_6_ node and FeCO site) for each of the two different regions that is different from the thermal component. The second RSVs of the FeCO site and Zr_6_ node both increase at around time zero, followed by a subsequent decrease over time. While the second RSV of the FeCO site exhibits only a weak oscillatory feature, the second RSV of the Zr_6_ node shows a strong oscillatory feature, which has a peak and a dip at around 1.35 and 3.35 ps, respectively. For a quantitative analysis, we globally fitted the two RSVs by modelling their time profiles as a convolution of an instrument response function (IRF) with a sum of three functions: exponential rise and decay, alongside a damped cosine function. During this global fitting process, the same time constants were shared by each function for the fitting of the two RSVs. The IRF was approximated by a Gaussian function with a full width at half maximum (FWHM) of 200 fs, reflecting a roughly estimated value for this experiment. A satisfactory fit was obtained with a time constant of 47.1 ± 0.5 ps for the exponential decay and two time constants of 5.55 ± 0.01 and 2.68 ± 0.02 ps for the period and damping time constant of the oscillatory motion, respectively.

### Kinetic modelling

**Fig. 3:**
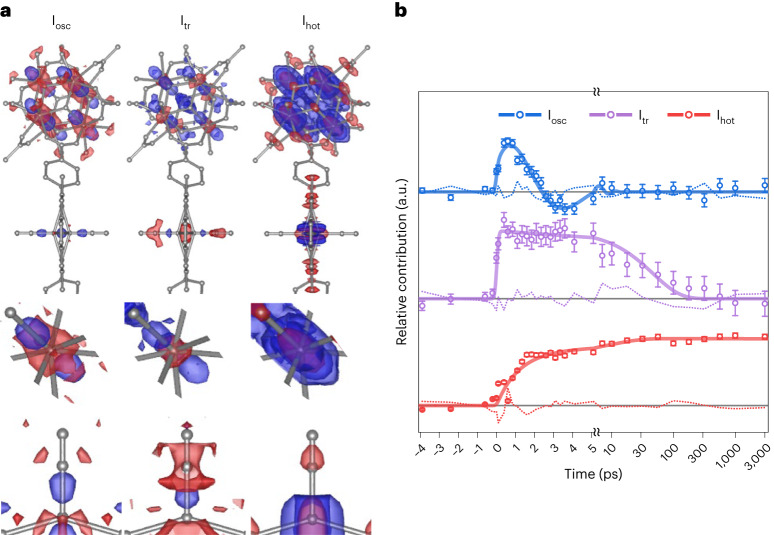
SADED maps and kinetics of three structural species. **a**, SADED maps of the oscillatory structure, I_osc_ (left), the transient structure, I_tr_ (centre), and the vibrationally hot structure, I_hot_ (right). The top panels show the SADED maps of the entire PCN–224(Fe)–CO, and the middle and bottom panels show the SADED maps of the Zr_6_ node and FeCO site, respectively. The red and blue colours show the negative and positive EDs, respectively, contoured at ±3*σ* (0.16 e Å^−^^3^ for I_osc_, 0.09 e Å^−^^3^ for I_tr_ and 0.43 e Å^−^^3^ for I_hot_) in the top and middle panels and contoured at ±2*σ* (0.1 e Å^−^^3^ for I_osc_, 0.06 e Å^−^^3^ for I_tr_ and 0.28 e Å^−^^3^ for I_hot_) in the bottom panels. Each *σ* represents the rms value calculated by taking the rms of the DED values for the corresponding SADED map. **b**, Time profiles of the SADED maps. The time-dependent contributions of the SADEDs of the three species (circles) to the DED maps at each time delay are fitted by the kinetic model described in the main text (solid line). The excellent agreement between the experimental data and the theoretical fit (dotted line) proves the validity of the kinetic model. Note that the size of the error bars is increased 50-fold to increase the visibility. The time axis scales are linear up to 5 ps and logarithmic thereafter. I_osc_ shows an oscillation with a period of 5.55 ps and damping time constant of 2.68 ps. I_tr_ appears within 200 fs and decays with a time constant of 47.1 ps. I_hot_ rises with time constants of 1.143 and 11.32 ps. Source data

Using the obtained time constants, we constructed a kinetic model that matches the behaviour of the major RSVs. The kinetic model consists of three structural species: a species corresponding to the oscillatory motion (I_osc_) with a period of 5.55 ps and a damping constant of 2.68 ps, a second species corresponding to the transient structure (I_tr_), which forms instantaneously (within the IRF of the experiment) upon photoexcitation and decays with a time constant of 47.1 ps, and a third species corresponding to the vibrationally hot structure (I_hot_), which persists up to 3 ns. Through a kinetic analysis using the kinetic model, we extracted the species-associated DED (SADED) maps corresponding to each of these species. The entire DED maps of PCN–224(Fe)–CO were used in this procedure, rather than the maps of just the regions around the Zr_6_ node or FeCO site. The SADED maps are shown in Fig. 3a. The characteristic feature in the SADED map of I_hot_ is the strong negative densities at the original positions of FeCO and the Zr atoms surrounded by strong isotropic positive densities, as revealed in the time-resolved DED maps shown in Fig. 2. By analysing the temporal profile of the SADED for I_hot_, we found that the thermal contribution rises with two exponential time constants of 1.143 ± 0.005 and 11.32 ± 0.07 ps (Fig. 3b). In contrast to the isotropic positive densities observed for I_hot_, the SADED map of I_tr_ exhibits distinct anisotropic features. In the ground state, the Zr atom is inherently disordered in two adjacent positions, with the axis connecting them defined as the *d* axis. I_tr_ exhibits a pair of strong positive densities at peripheral positions along this axis. This implies that the disorder of the Zr atoms is further enhanced along the *d* axis in I_tr_. The Fe porphyrin shows a strong negative feature at the position of the CO ligand, consistent with the photodissociation of CO. In addition, a positive feature is observed between the original Fe and C atom positions, and a negative feature is visible below the original Fe atom position, indicating that the Fe atom has moved away from the haem centre. In other words, the porphyrin already domed (exhibiting an out-of-plane displacement) in the ground state undergoes more intense doming in I_tr_. Finally, the SADED map of I_osc_ shows similar features to the SADED map for I_tr_ in the Zr_6_ node, whereas the FeCO site shows distinct features: a negative density is evident at the original position of the Fe atom, with two strong positive densities above and below the negative density along the axis connecting the Fe and CO ligand. These features indicate that the structural identity of I_osc_ stems from the oscillation of the Zr atoms in the Zr_6_ node along the *d* axis and the simultaneous oscillation of the Fe atom in the Fe porphyrin along the axis connecting the CO ligand.

### Structural analysis using extrapolated maps

**Fig. 4:**
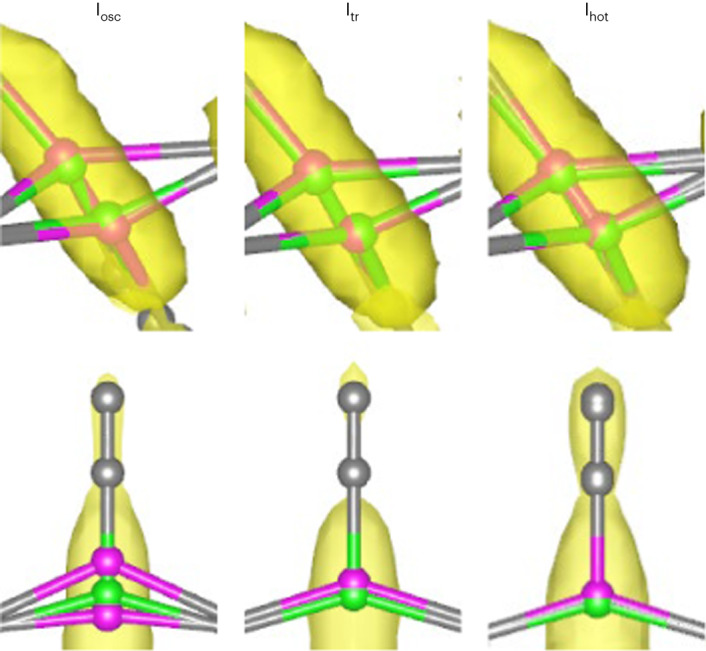
Structural analysis of the three structural species. The extrapolated maps of the three species I_osc_, I_tr_ and I_hot_ for the Zr_6_ node region (top) and FeCO site (bottom). The extrapolated maps were generated using the SADED maps shown in Fig. 3, the ED map of the ground-state structure and the photoconversion yield. Specifically, an extrapolated map was obtained for each species by refining the structure factors that were generated through a linear combination of the structure factors derived from the corresponding SADED and ground-state ED maps. The photoconversion yield of each species was extrapolated to 100%, meaning that each species exists in a pure form. The details of the generation of the extrapolated maps and subsequent structure refinement to obtain the structures of the three species are reported in Methods. The yellow isosurfaces are contoured at 2*σ* (1.5 e Å^−^^3^ for I_osc_, 1.3 e Å^−^^3^ for I_tr_ and 1.3 e Å^−^^3^ for I_hot_). Each *σ* represents the rms value calculated by taking the rms of the ED values for the corresponding extrapolated map. The green and magenta spheres represent the positions of both the metal atoms Zr and Fe in the ground-state structure and the three structural species, respectively. The viewing perspective of the Zr_6_ node region is altered compared with the viewing perspective shown in Fig. 3 for better visualization, whereas that of the FeCO site is identical.

To extract the structures of the three species from the SADEDs, the extrapolated structure factors (*F*_extr_) were obtained from the following three factors: the photoconversion yields, the structure factors of the SADEDs and the structure factors of the ground state (Methods). We have the structure factors (*F*) when the conversion yield is 0, which is the structure factor of the ground-state structure, and the species-associated difference structure factor amplitudes (Δ|*F*|_SA_; obtained by inverse Fourier transformation of the SADED), which corresponds to the actual conversion yield. With these two values as a starting point, we can determine *F*_extr_, corresponding to the conversion yield of unity, through linear extrapolation. The estimated *F*_extr_ values represent the structure factors of the structural species. We obtained the atomic structures of the structural species from *F*_extr_ by applying structure refinement to the square of *F*_extr_. The refined structures and the corresponding EDs of the extrapolated maps are presented in Fig. 4. The crystallographic data are provided in Extended Data Table 1 and the key structural parameters are summarized in Table 1. A distinguishing characteristic of the three species is the position of the Fe atom. Compared with the ground state, the Fe atom in the I_tr_ structure shows an increase in doming of 0.119 Å. This enhanced doming of I_tr_ compared with the ground state is consistent with previously reported structures of Fe tetraphenylporphyrin, which is similar to the Fe porphyrin building block in the PCN–224(Fe) MOF. Previous studies have shown that the degree of doming is influenced by the coordination number of the Fe atom^33,34^. In the presence of a CO ligand, the pentacoordinate Fe atom is displaced out of the plane by ~0.2 Å. In the absence of any bound axial ligands, the tetracoordinate Fe atom is displaced out of the plane by ~0.4 Å, indicating an additional doming of 0.2 Å compared with the displacement with CO binding. The observed enhanced doming of 0.119 Å for I_tr_, occurring together with CO photodissociation, agrees with the reported 0.2 Å increase in doming for the tetracoordinate geometry of the Fe atom. In the I_osc_ structure, the Fe atom seems to oscillate vertically from the original position (approximately 0.443 Å upwards and 0.312 Å downwards). The I_hot_ structure resembles the ground state, with only minor variations in the bond lengths. For the Zr_6_ node, the Zr atoms of all three species are moved out further along the *d* axis, with distinct Zr–Zr distances ranging from 0.891 to 1.057 Å. The observed structural changes in the Zr_6_ node correlate with the out-of-plane doming motion of the Fe atom, demonstrating a strong positive correlation between the Zr–Zr distance and the degree of Fe doming (Table 1). Both the kinetics and SADED maps of the three structural species are generally consistent with those of myoglobin, one of the most well-studied Fe porphyrin-containing systems, but a number of differences are also observed, as detailed in Supplementary Discussion.

**Table 1:** Comparison of the photoconversion yields, structural parameters and *R* factors for the three structural species obtained from the extrapolated map analysis

Structural species	*p* (%)	Fe doming (Å)		Zr–Zr distance (Å)		*R*_*1*_ (%)
Ground state		0.597(6)		0.841(11)		13.41
I_osc_	25	1.040(3) 0.285(2)	+0.443 −0.312	1.057(7)	+0.216	13.99
I_tr_	20	0.716(7)	+0.119	0.907(10)	+0.066	11.88
I_hot_	100	0.684(4)	+0.087	0.891(7)	+0.050	7.74

The photoconversion yields (*p*) used to generate the extrapolated maps, the structural parameters (Fe doming and Zr–Zr distance) derived from the refinement of the extrapolated maps and the corresponding *R*_*1*_ factors, which represents the discrepancy between observed and calculated structure factors, are listed. The parameters for the ground state are also included for comparison. The values in parentheses are standard deviations. The values in the right-hand columns of Fe doming and Zr–Zr distance are the differences from the ground-state values. The Fe doming value was calculated as the distance of the Fe atom from the plane formed by the four carbon atoms connecting the pyrrole groups in the TCPP ligand.

### Structural dynamics

**Fig. 5:**
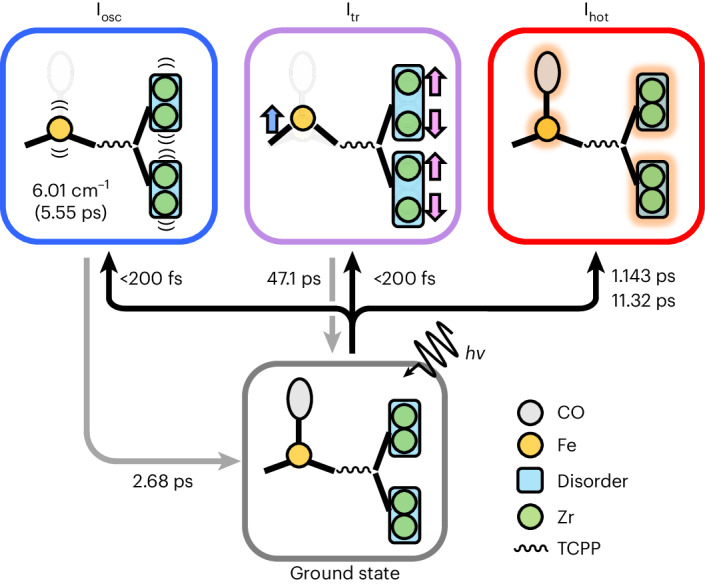
Photoinduced trifurcating structural changes of PCN–224(Fe)–CO observed via TR-SFX. Upon photoexcitation, a CO molecule dissociates from FeTCPP and organized anisotropic movements occur within the instrument response function (<200 fs). The organized anisotropic movements include coherent oscillation (I_osc_) and the formation of a transient structure (I_tr_). In I_osc_, the Fe and Zr atoms oscillate with a period of 5.55 ps. The Fe atom oscillates in a doming–undoming manner along the direction perpendicular to the plane formed by TCPP, while the Zr atoms oscillate along the *d* axis connecting two different occupation sites of a Zr atom due to positional disorder. In I_tr_, the doming of the porphyrin (blue arrow) and the disordering of the Zr atoms (magenta arrows) in the Zr_6_ node are enhanced. I_osc_ and I_tr_ decay with time constants of 2.68 and 47.1 ps, respectively. The structure of the ground state (faint lines) is superimposed on the schematic images of I_osc_ and I_tr_ for comparison. In addition to the organized anisotropic movements, the vibrationally hot structure (I_hot_) with isotropic structural movement is formed with time constants of 1.143 and 11.32 ps. In I_hot_, Zr, Fe and the CO ligand all exhibit broader electron distributions (orange shading) than in the ground state due to vibrational excitation. Zr, Fe and the CO ligand are shown in green, orange and grey, respectively. The TCPP ligand connecting the Zr and Fe sites is represented by a wavy line. The two disordered positions of a Zr atom in the ground state and each structural species are represented in cyan. *h*ν represents the photon energy, where *h* is Planck’s constant and ν is the frequency of the photon.

Figure 5 schematically illustrates the trifurcating structural pathways unveiled in this study. As the Zr_6_ node does not absorb 400 nm photons, the transient disordering motions of the Zr atoms must be caused by the photon-absorbing Fe porphyrin sites. Thus, the organized anisotropic movement of the Fe porphyrin and Zr_6_ node observed at early time delays implies that the structural perturbation caused by the dissociation of the CO ligand from the Fe atom is instantly transferred to the Zr_6_ node, causing organized structural changes in the Zr_6_ node. Such movements exhibit even coherent oscillations in both the Zr_6_ node and FeCO in the Fe porphyrin. We note that an oscillatory component has rarely been visualized directly in the form of a DED map in previous studies with time-resolved crystallography. The large number of time points and high signal-to-noise ratios of the data acquired in this work were critical to capturing this DED feature. The oscillatory motion has a period of 5.55 ps, which corresponds to 6.01 cm^−1^ or 0.18 THz. The SADED map of I_osc_ associated with oscillation at 0.18 THz exhibits organized movement at the photon-absorbing porphyrin site and remote zirconium site. Such a slow oscillatory motion is rarely observed in small molecules, but is feasible as a phonon mode in a solid sample. In general, the low-frequency phonon modes of MOFs originate from the acoustic phonon modes related to the motion of the ‘chain’ between the node and the linker^35^^,36^. Theoretical studies reported that the transverse or longitudinal acoustic phonons of MOFs directed along the *Γ*–*X* axis, corresponding to the direction connecting ligands and metal clusters, exhibit frequencies below 0.3 THz^35,37^. The observed features in the SADED map of I_osc_ are not consistent with those of acoustic photon modes. For example, according to Fig. 3, of the four O atoms connected to each Zr atom in the Zr_6_ node (Fig. 1a), the change in the DED of Zr is directed towards the O atom that lies on the *d* axis rather than along the axis connecting the Fe porphyrin and Zr_6_ node. In addition, Fe in the linker porphyrin shows a doming motion, which corresponds to a bending motion in the out-of-plane direction. These motions are different from the low-lying acoustic phonons of MOFs. According to the theoretical study^35^, similar motions correspond to the lowest-energy optical phonon of a MOF with Mg nodes with a frequency of 0.57 THz, although this is considerably higher than that observed in this work. Meanwhile, this theoretical study also suggested that the phonon frequency can decrease as the metal node becomes heavier. Considering this, the lower frequency (0.18 THz) of the optical phonon mode observed for PCN–224(Fe)–CO can be attributed to the heavier Zr_6_ node than Mg and Ca clusters used in theoretical calculation^35^. Based on these considerations, the DED feature observed for I_osc_ originates from the optical phonon mode activated by photoexcitation rather than acoustic phonons. Later, the vibrationally hot structure with isotropic features (I_hot_) comes to dominate over time. It should be noted that DED maps from such vibrationally hot structures are rarely observed in protein crystals. The lack of vibrationally hot features in protein crystals is probably due to the presence of water and buffer molecules in the protein crystals, which effectively absorb the heat generated by the absorption. In summary, the time-resolved DED maps reveal three distinct structural pathways: at early time delays, both Fe and Zr atoms undergo coherent oscillations as well as transient structural changes, while at later time delays, they become disordered, displaying a vibrationally hot configuration.

We have demonstrated here that chemical systems can be studied with TR-SFX, which previously has been used only for protein crystals but has the potential to be applied to a wide range of porous materials and chemical systems.

## Methods

### Synthesis of PCN–224

Micro-sized crystals of PCN–224 were prepared for TR-SFX analysis following a reported procedure^38^ with a slight modification. All of the chemicals required for the preparation of the MOF crystals were purchased from commercial suppliers (Sigma Aldrich and TCI Korea) and used as received without further purification. For the synthesis of the micro-sized crystals, ZrCl_4_ (0.21 g, 0.90 mmol; Sigma Aldrich, 99.5%), TCPP (0.070 g, 0.088 mmol; TCI Korea, 97.0%), benzoic acid (3.66 g, 30 mmol; Sigma Aldrich, 99.5%) and acetic acid (3.4 ml, 60 mmol; Sigma Aldrich, 99.7%) were suspended in *N*,*N*-dimethylformamide (DMF; 14 ml) in air in a 30 ml vial with a Teflon-lined cap and heated at 130 °C for 3 days to give red-brown cubic crystals. The vial was cooled for 1 h and the mother liquor was decanted. The remaining crystals were washed with DMF (10 × 10 ml) for 2 days and then with dichloromethane (DCM; 10 × 10 ml) for a further 2 days. To remove any solvents absorbed inside the pores of the resulting PCN–224, the washed crystals were heated at 150 °C for 12 h under vacuum. The characterization of PCN–224 is described in detail in Supplementary Methods.

### Synthesis of PCN–224(Fe^II^) and PCN–224(Fe^II^)–CO

FeCl_2_ (558.0 mg, 4.4 mmol; Sigma Aldrich, 98%) and 2,6-lutidine (102.5 μl, 0.880 mmol; Sigma Aldrich, 97%) were mixed in DMF (5 ml) in a 25 ml round-bottomed flask. PCN–224 (150 mg, 0.037 mmol) was then immersed in this solution and heated for 12 h at 150 °C. After the reaction, the supernatant was decanted and the remaining crystals were washed in DMF (3 × 10 ml) for 30 min at 150 °C and then decanted. Next, 10 ml DMF was added to the washed crystals in the flask, which was then sealed and sonicated for 30 min. These crystals were washed with DMF (10 × 10 ml) and DCM (10 × 10 ml) for 2 days each. The remaining crystals were then dried for 12 h at 150 °C under vacuum, yielding dark-purple crystals. Unless otherwise noted, these procedures were conducted under N_2_ atmosphere using a Schlenk line or under Ar atmosphere in a glove box. After the activation of PCN–224(Fe^II^), PCN–224(Fe^II^) was exposed to 1 atm CO for 4 h to generate PCN–224(Fe^II^)–CO.

### Sample preparation for TR-SFX

Freshly activated PCN–224(Fe^II^) was transported as a dry solid encapsulated in Ar to the Pohang Accelerator Laboratory X-ray free-electron laser (PAL-XFEL). A fixed-target sample holder was especially designed to keep the sample exposed to CO during the experiment. A fixed-target sample holder has two major advantages over conventional injector-style crystal delivery systems. First, the amount of sample consumed in the experiment can be greatly reduced. Second, there are no problems of chemical compatibility of the sample crystals with the medium because the crystals are simply placed on a substrate, such as a thin film, rather than dispersed in a liquid or gel-type medium. Acrylic Kapton tape was placed on one side of the sample holder and activated PCN–224(Fe^II^) crystals were spread over the Kapton tape under an Ar atmosphere. Then, 50 μm polypropylene film was attached to the other side of the holder using double-sided acrylic tape. Samples encapsulated in the holder were exposed to ~1 atm CO for 4 h to generate PCN–224(Fe^II^)–CO. After the exposure, the holder was completely sealed and subjected to TR-SFX analysis.

### TR-SFX data collection

The diffraction experiments were performed at the XSS beamline of PAL-XFEL. The PAL-XFEL delivered X-ray pulses with a photon energy of 14.5 keV at a repetition rate of 30 Hz. The X-ray beam was focused to a spot of ~11 μm diameter full-width at half-maximum (FWHM). Laser pulses with a wavelength of 800 nm were generated from a Ti:sapphire regenerative amplifier and then converted to 400 nm pulses with a duration of <100 fs. The laser beam was focused to a spot of 45 μm diameter FWHM with a laser fluence of 5 mJ mm^−^^2^. Large-diameter X-ray and laser beams were used to prevent damage to the film and tapes and to keep the sample sealed in the CO environment. The X-ray and laser beams were aligned to overlap at the sample position with a crossing angle of 10°. The samples were delivered in home-made fixed-target sample holders mounted on Kohzu XA05A-L202 stages and moved for raster scanning. Time-resolved diffraction data were collected at 33 time delays between X-ray and laser pulses: −3.9, −2.4, −0.6, −0.2, 0.1, 0.4, 0.6, 0.85, 1.1, 1.35, 1.6, 1.85, 2.1, 2.35, 2.6, 2.85, 3.1, 3.35, 3.6, 4.1, 5.1, 7.1, 10.1, 17.9, 31.7, 56.3, 100, 178, 316, 562, 1,000 and 3,000 ps. More than 15,000 diffraction images were collected at each time delay. The diffraction images were recorded with a Rayonix MX225-HS detector in 4 × 4 binning mode (pixel size: 156 μm × 156 μm).

### Data processing and analysis

We used the Cheetah software^39^ to select images containing meaningful diffraction spots by filtering out empty images and find spot positions in the selected diffraction images. Around 10–25% of the images collected from the sample at each time delay were identified as containing Bragg spots (Supplementary Tables 1–5).

The hit images obtained were further indexed and integrated using CrystFEL^40^. For indexing, the XGANDALF algorithm provided by CrystFEL was used, and the data beyond 0.9 Å were neglected due to the large experimental noise in the high-resolution region. The parameters for the experimental geometry were optimized by a trial-and-error approach. Intensities were integrated using the ‘rings-sat-cen’ option in CrystFEL with concentric rings of three, five and six pixels for the peak, buffer and background regions, respectively. The 'rings-sat-cen' integration option (i) utilizes three concentric rings for delineating peak and background areas, (ii) centers peak boxes on actual peak locations iteratively and (iii) does not integrate reflections that contain saturated pixels. The integrated intensities were merged using the partialator program from CrystFEL. XPREP was used to initially determine the space group of the crystal and prepare an input file for solving the structure. X-ray absorption was found to be negligible for crystals in the size range of 10–20 µm, where *T*_min_, the minimum value for experimental X-ray absorption correction, is larger than 0.9. Consequently, no absorption correction was applied. EDs and the corresponding molecular structures were solved using SHELXT^41^. VESTA^42^ was used to visualize ED maps overlain on refined molecular structures.

The space group of PCN–224(Fe)–CO is the same as that of a previously reported crystal structure of PCN–224(Fe) obtained by single-crystal X-ray crystallography^38^. The structure at each time delay, solved using SHELXT, was refined by a full-matrix least-squares method on *F*^2^ using SHELXL^43^. All non-hydrogen atoms were refined anisotropically and hydrogen atoms were refined isotropically. The structures were refined by applying displacement parameter restraints using DELU and SIMU commands and by applying a distance restraint using DFIX command in SHELXL. The disorder around the Zr clusters in the structures was modelled at each time delay. We observed a substantial residual ED in the ED maps that cannot be accounted for by the refined structures. This residual density may be attributed to either solvent molecules remaining in the pores or the partial occupation of MOF-525, which has a similar composition to PCN–224, but possesses a distinct structure. We employed the SQUEEZE option in PLATON^44^ to model the residual ED as contributions from disordered solvents or gas within the voids. The results of the refinement are summarized in Supplementary Tables 1–5. The A/B alerts generated from running CheckCIF and explanations are summarized in Supplementary Table 6. As the ORTEP-style drawings of the asymmetric unit of PCN–224(Fe)–CO at all time delays are very similar, we have included only the illustration corresponding to the laser-off condition as an example (Supplementary Fig. 1).

The experimental structure factor amplitudes (|*F*|) at each time delay were scaled based on the calculated (absolute) amplitudes. Δ|*F*| values were then obtained by subtracting the reference structure factor amplitude from the structure factor amplitudes at each time delay, represented as Δ|*F*|(*hkl*, *t*) = |*F*(*hkl*, *t*)| − |*F*(*hkl*, reference)|. Here, *hkl* represents the Miller indices and *t* denotes the time delay. The reference structure factor amplitude was derived from the average of the structure factor amplitudes measured at the negative time delays (−3.9, −2.4, −0.6 and −0.2 ps). DED (Δ*ρ*) maps were generated by Fourier transformation of each set of difference structure factor amplitudes using the phases from the PCN–224(Fe)–CO model before reaction^45–47^. Extended Data Figs. 1 and 2 show the DED maps at all time delays contoured at ±5.0*σ* (0.58 e Å^−^^3^) and ±3.0*σ* (0.35 e Å^−^^3^), respectively. Instead of a whole DED map, two regional DED maps, which are expected to arise from large structural changes, were used for further analysis: one was around the Fe atom and its CO ligand in the porphyrin ring (Extended Data Fig. 3) and the other was around the Zr_6_ node (Extended Data Fig. 4). In the DED maps obtained at late time delays, the original metal positions have strong negative densities surrounded by strong positive densities. Such features indicate that the metal atoms have been displaced from their original positions to other positions in random orientations, which is a characteristic of vibrationally hot structures. To confirm this hypothesis, the DED maps corresponding to the vibrationally hot structure, I_hot_, were simulated for comparison with the experimentally observed DED maps. For the simulation, we calculated the ED maps for PCN–224(Fe)–CO before and after thermal excitation. For the ED map before thermal excitation, we used the ED map of the refined structure of laser-off PCN–224(Fe)–CO, which was obtained with the excitation laser off. To generate the ED maps of Zr and Fe atoms after thermal excitation, we increased the atomic displacement parameter of each atom in the refined structure of laser-off PCN–224(Fe)–CO and calculated the corresponding ED maps. The DED maps were obtained by subtracting the ED map before thermal excitation from that after thermal excitation (Extended Data Fig. 5). The simulated DED maps obtained on the assumption that the atomic displacement parameters increase due to the heating of the MOF have similar features to the experimental DED maps at late time delays (Extended Data Fig. 5). Based on these results, DED maps at late time delays were assigned to the heating component (that is, the vibrationally hot structure). Heat-free DED maps were obtained by removing the feature corresponding to the heating component from the DED map at each time delay (Supplementary Fig. 2).

### Kinetic analysis of TR-SFX data

Data were collected at 33 time delays, which provides a large enough dataset for a quantitative kinetic and structural analysis. To obtain quantitative kinetic information from time-resolved DED maps, we applied SVD to decompose the original data into time-invariant DED features (that are, LSVs), their relative contributions (singular values) and their time profiles (that is, RSVs). First, the DED maps around the Zr_6_ node and the Fe porphyrin site were separately decomposed by SVD analysis to emphasize the dynamics of each metal domain. The singular values, autocorrelation values, and major LSVs and RSVs are shown in Extended Data Fig. 6. Inspection of the singular values and vectors revealed that, in both regions, the first component (the first LSVs and RSVs) stands out. The first RSVs for the two regions have almost identical shapes (Extended Data Fig. 7a). In addition to this dominant component, additional major components (the second RSVs) can also be observed in both regions.

For the primary component of both the Zr_6_ node and FeCO site, the laser-off positions of Zr and Fe exhibit strong negative densities surrounded by an isotropic shell of positive densities. This feature represents a vibrationally hot structure that extends through both metallic sites. Accordingly, the first RSVs represent the kinetics of the vibrationally hot structure, that is, thermal kinetics. To confirm this, we also performed a modified analysis using the Wilson plot method^48^. The Wilson plot was used to estimate the difference between the scale factors and the overall *B*-factors between datasets. The structure factors rescaled from 0 to 0.25 Å^−2^ during the structure refinement using SHELXL were used to extract the difference between the *B*-factors (Extended Data Fig. 7b). Specifically, we extracted the difference between the *B*-factor at each time delay and that of the averaged structure factors at negative time delays and compared the temporal trend with the first RSVs (Extended Data Fig. 7a). The first RSVs and the difference *B*-factors showed excellent agreement, indicating that the first RSV represents thermal kinetics. As the first RSVs for the two regions have nearly identical shapes (Extended Data Fig. 7a), they represent the kinetics of thermal excitation of the entire PCN–224(Fe) structure following photoexcitation. In this regard, we conducted a quantitative analysis of the time-resolved temperature changes in PCN–224(Fe); details of the analysis are provided in Supplementary Discussion and the results are presented in Supplementary Fig. 10.

For the Zr_6_ node, the second LSV exhibits an anisotropic feature along the *d* axis connecting the two different occupation sites of a Zr atom arising from positional disorder. Along the *d* axis, a negative density appears between the two occupation sites of a Zr atom, and two positive densities emerge at the exterior of the two occupation sites. This indicates that the Zr atoms were further disordered along the *d* axis. The components ranked third and below have low autocorrelation and singular values, and hence their contributions to the time-resolved DED maps around the Zr_6_ node can be ignored. For the FeCO site, the second LSV also shows clear anisotropic features along the axis connecting Fe and the CO ligand. Here, a pair of strong positive and negative densities appear above and below the original position of the Fe atom, indicating that this LSV represents the directional movement of the Fe atom. In addition, a strong negative density is evident around the position of the CO ligand, indicating that it dissociates upon photoexcitation.

**Extended Data Fig. 8:**
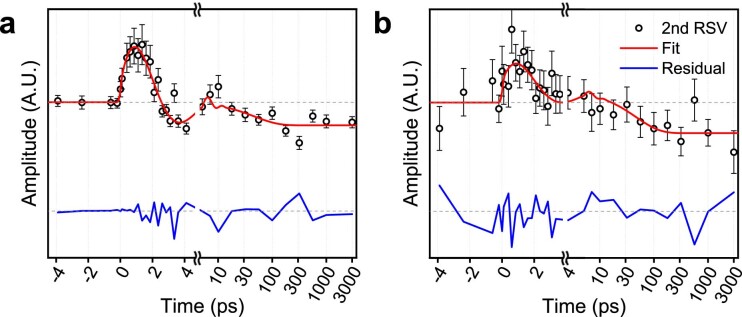
Kinetic analysis results of the second RSVs obtained from the SVD analysis on the DED maps of the Zr_6_ node and FeCO site. The 2nd RSV of the DED maps and the fits for (**a**) the Zr_6_ node and (**b**) the FeCO site. Two RSVs were globally fitted. Those RSVs (black) were fitted using an exponential decay with a time constant of 47.1 ps and a damped cosine with a period of 5.55 ps and a damping time constant of 2.68 ps. The fits and residuals are shown in red and blue, respectively. The error bars are magnified by a factor of 50 to increase visibility. Source data

The characteristic features of the RSVs can be briefly inspected before quantitative analysis. The second RSV for the Zr_6_ node shows three distinct behaviours: instantaneous rise, oscillation and exponential decay. As shown in Extended Data Fig. 6, the second RSV shows an instantaneous rise at around time zero, indicating that the contribution of the second LSV rises immediately upon photoexcitation. After the rise at time zero, the second RSV shows substantial oscillatory features with a peak at around 1.25 ps and a dip at around 4.0 ps. This oscillatory behaviour does not persist to late time delays, but damps quickly. Finally, the second RSV decays slowly towards zero. The second RSV of the FeCO site exhibits characteristics similar to those observed for the second RSV of the Zr_6_ node, particularly the instantaneous rise and exponential decay behaviours. However, the oscillatory behaviour of the second RSV of the FeCO site is notably weaker than that observed for the Zr_6_ node. Nevertheless, on closer examination, it is evident that these oscillatory features still make a discernible contribution. We performed a kinetic analysis for the two meaningful RSVs, the second RSVs of the Zr_6_ node and the FeCO site, to extract the detailed kinetics of the photoinduced anisotropic structural changes. For a quantitative analysis, the two second RSVs were globally fitted as a convolution of the IRF of ~200 fs FWHM with the sum of an exponential rise function (with a time constant of 20 fs, representing an instantaneous rise within the IRF of the experiment), an exponential decay function and a damped cosine function sharing time constants. The results of the fit are shown in Extended Data Fig. 8. A satisfactory fit was obtained with a time constant of 47.1 ± 0.5 ps for the exponential decay and two time constants of 5.55 ± 0.01 and 2.68 ± 0.02 ps for the period and damping, respectively, of the oscillatory motion.

Using the obtained time constants, we constructed a kinetic model that matches the behaviour of the major RSVs. The kinetic model consists of three species: (1) a species corresponding to the vibrationally hot structure (I_hot_), (2) a species corresponding to the transient structure (I_tr_) that forms instantaneously within the IRF of the experiment upon photoexcitation and decays with a time constant of 47.1 ps, and (3) a species corresponding to the oscillatory motion (I_osc_) with a period of 5.55 ps and a damping constant of 2.68 ps. The DED maps corresponding to these three species (that is, the SADED maps) were extracted via a kinetic analysis using the kinetic model. In this procedure, the entire DED maps of PCN–224(Fe)–CO were used instead of maps of just the regions around the Zr_6_ node and FeCO site. The kinetic analysis performed using the kinetic model is described in detail in Supplementary Methods. The resulting SADED maps for I_osc_, I_tr_ and I_hot_ and their time profiles are shown in Fig. 3a,b.

### Generation of extrapolated maps

Detailed structural parameters for the structural species were obtained by structure refinement. The structure refinement was performed on diffraction intensities represented by the square of the structure factors of each structural species. The structure factors of the structural species, the so called extrapolated structure factors (*F*_extr_(*hkl*))^49^, were obtained as follows: first, species-associated difference structure factors (Δ|*F*|_SA_(*hkl*)) for each structural species were obtained through inverse Fourier transformation of the corresponding SADEDs, derived from the kinetic analysis. The structure factors of the laser-off structure were used as the structure factors for ground state (*F*_Ground_(*hkl*)). Then, the amplitudes of *F*_extr_(*hkl*), |*F*|_extr_(*hkl*), were computed as follows:

1 $$|F{|}_{{\rm{extr}}}(hkl)=|F{|}_{{\rm{Ground}}}(hkl)+(1/p)\times \,\varDelta |F{|}_{{\rm{SA}}}(hkl)$$

where |*F*|_Ground_(*hkl*) is the amplitude of *F*_Ground_(*hkl*), *p* is the photoconversion yield ranging from 0 to 1 and Δ|*F*|_SA_(*hkl*) is the amplitude of Δ*F*_SA_(*hkl*). By determining the optimal value of *p*, |*F*|_extr_(*hkl*), free of the contribution of the ground state can be obtained. Suitable indicators for evaluating the level of photoconversion include the absence of negative density on the Zr atoms. As is evident from the term containing the division of Δ*F*_SA_(*hkl*) by *p* on the right-hand side of equation (1), it should be noted that when *p* is equal to unity (that is, when all of the ground state has been converted into the structural species), |*F*|_extr_(*hkl*) is simply the sum of the amplitudes of |*F*|_Ground_(*hkl*) and Δ|*F*|_SA_(*hkl*). When *p* is not equal to unity, the difference, Δ|*F*|_SA_(*hkl*), is weighted and added to |*F*|_Ground_(*hkl*) to obtain |*F*|_extr_(*hkl*). This process is referred to as extrapolation because typically, for *p* values less than 1, the small difference structure factors are extrapolated (or amplified to correspond to *p* = 1) and added to the structure factor of the ground state to determine the structure factors of the newly formed species. The *p* factors were determined to be 1.0 for I_hot_, 0.20 for I_tr_ and 0.25 for I_osc_. Next, we calculated |*F*|_extr_(*hkl*) using the obtained |*F*|_extr_ and the phase of the laser-off structure. The resulting |*F*|_extr_(*hkl*) values were squared to generate the diffraction intensities of the structural species. The diffraction intensities were refined in a similar manner to that used for the refinement of the laser-off structure, with additional restraints on the distances and planarity of the phenyl and pyrrole groups in the TCPP ligand. The A/B alerts associated with the structural species and the corresponding explanations are summarized in Supplementary Table 7.

The *R* factors for the extrapolated maps of the three structural species are comparable to, or even smaller than, those of the static crystal structures shown in Supplementary Tables 1–5. These small *R* factors for the structural species can be attributed to the use of a large number of DED maps for the calculation of the SADED maps. A SADED map can be described as a linear combination, or weighted average, of the DED maps, as detailed in Supplementary Methods. Due to this averaging process, in which the noise from each DED map is offset against one another, the noise in the SADED maps is reduced compared with that in individual DED maps. Consequently, SADED maps with reduced noise result in high-quality |*F*|_extr_, eventually leading to reduced *R* factors for the extrapolated maps. For instance, we can compare the quality of Δ|*F*|_SA_ for I_hot_ and Δ|*F*| at 1 ns. At time delays much longer than the decay time constant of I_tr_ (47.1 ps), only I_hot_ contributes to Δ*F*. Because *p* is equal to unity, it can be inferred that Δ|*F*| values at late time delays are approximately equivalent to Δ|*F*|_SA_ for I_hot_. In simple terms, the information from Δ|*F*| at multiple late time delays is averaged to extract Δ|*F*|_SA_ for I_hot_. Consequently, the quality of Δ|*F*|_SA_ is inherently improved compared with Δ|*F*| for a single time delay, such as 1 ns. As Δ|*F*|_SA_ of I_hot_ would be of higher quality than that of Δ*F* at 1 ns, it is reasonable to obtain a smaller *R* factor for the extrapolated map of I_hot_ (7.33%) than for the crystal structure at 1 ns (10.9%).

## Online content

Any methods, additional references, Nature Portfolio reporting summaries, source data, extended data, supplementary information, acknowledgements, peer review information; details of author contributions and competing interests; and statements of data and code availability are available at 10.1038/s41557-024-01460-w.

### Supplementary information

Supplementary Information Supplementary Methods, Discussion, Figs. 1–10 and Tables 1–8.

Supplementary Data 1 Crystallographic structure factors for PCN–224(Fe)–CO at laser off; CCDC reference number 2308176.

Supplementary Data 2 Crystallographic structure factors for PCN–224(Fe)–CO at −3.9 ps; CCDC reference number 2308180.

Supplementary Data 3 Crystallographic structure factors for PCN–224(Fe)–CO at −2.4 ps; CCDC reference number 2308179.

Supplementary Data 4 Crystallographic structure factors for PCN–224(Fe)–CO at −0.6 ps; CCDC reference number 2308178.

Supplementary Data 5 Crystallographic structure factors for PCN–224(Fe)–CO at −0.2 ps; CCDC reference number 2308184.

Supplementary Data 6 Crystallographic structure factors for PCN–224(Fe)–CO at 0 ps; CCDC reference number 2308177.

Supplementary Data 7 Crystallographic structure factors for PCN–224(Fe)–CO at 0.1 ps; CCDC reference number 2308185.

Supplementary Data 8 Crystallographic structure factors for PCN–224(Fe)–CO at 0.4 ps; CCDC reference number 2308183.

Supplementary Data 9 Crystallographic structure factors for PCN–224(Fe)–CO at 0.6 ps; CCDC reference number 2308181.

Supplementary Data 10 Crystallographic structure factors for PCN–224(Fe)–CO at 0.85 ps; CCDC reference number 2308182.

Supplementary Data 11 Crystallographic structure factors for PCN–224(Fe)–CO at 1.1 ps; CCDC reference number 2308207.

Supplementary Data 12 Crystallographic structure factors for PCN–224(Fe)–CO at 1.35 ps; CCDC reference number 2308208.

Supplementary Data 13 Crystallographic structure factors for PCN–224(Fe)–CO at 1.6 ps; CCDC reference number 2308210.

Supplementary Data 14 Crystallographic structure factors for PCN–224(Fe)–CO at 1.85 ps; CCDC reference number 2308209.

Supplementary Data 15 Crystallographic structure factors for PCN–224(Fe)–CO at 2.1 ps; CCDC reference number 2308205.

Supplementary Data 16 Crystallographic structure factors for PCN–224(Fe)–CO at 2.35 ps; CCDC reference number 2308202.

Supplementary Data 17 Crystallographic structure factors for PCN–224(Fe)–CO at 2.6 ps; CCDC reference number 2308203.

Supplementary Data 18 Crystallographic structure factors for PCN–224(Fe)–CO at 2.85 ps; CCDC reference number 2308201.

Supplementary Data 19 Crystallographic structure factors for PCN–224(Fe)–CO at 3.1 ps; CCDC reference number 2308204.

Supplementary Data 20 Crystallographic structure factors for PCN–224(Fe)–CO at 3.35 ps; CCDC reference number 2308206.

Supplementary Data 21 Crystallographic structure factors for PCN–224(Fe)–CO at 3.6 ps; CCDC reference number 2308220.

Supplementary Data 22 Crystallographic structure factors for PCN–224(Fe)–CO at 4.1 ps; CCDC reference number 2308218.

Supplementary Data 23 Crystallographic structure factors for PCN–224(Fe)–CO at 5.1 ps; CCDC reference number 2308222.

Supplementary Data 24 Crystallographic structure factors for PCN–224(Fe)–CO at 7.1 ps; CCDC reference number 2308221.

Supplementary Data 25 Crystallographic structure factors for PCN–224(Fe)–CO at 10.1 ps; CCDC reference number 2308217.

Supplementary Data 26 Crystallographic structure factors for PCN–224(Fe)–CO at 17.9 ps; CCDC reference number 2308215.

Supplementary Data 27 Crystallographic structure factors for PCN–224(Fe)–CO at 31.7 ps; CCDC reference number 2308214.

Supplementary Data 28 Crystallographic structure factors for PCN–224(Fe)–CO at 56.3 ps; CCDC reference number 2308213.

Supplementary Data 29 Crystallographic structure factors for PCN–224(Fe)–CO at 100 ps; CCDC reference number 2308216.

Supplementary Data 30 Crystallographic structure factors for PCN–224(Fe)–CO at 178 ps; CCDC reference number 2308219.

Supplementary Data 31 Crystallographic structure factors for PCN–224(Fe)–CO at 316 ps; CCDC reference number 2308057.

Supplementary Data 32 Crystallographic structure factors for PCN–224(Fe)–CO at 562 ps; CCDC reference number 2308056.

Supplementary Data 33 Crystallographic structure factors for PCN–224(Fe)–CO at 1 ns; CCDC reference number 2308055.

Supplementary Data 34 Crystallographic structure factors for PCN–224(Fe)–CO at 3 ns; CCDC reference number 2308058.

Supplementary Data 35 Crystallographic structure factors for the transient species; CCDC reference number 2308738.

Supplementary Data 36 Crystallographic structure factors for the oscillating species; CCDC reference number 2308736.

Supplementary Data 37 Crystallographic structure factors for the vibrationally hot species; CCDC reference number 2308737.

### Source data

Source Data Fig. 3 Statistical source data—Excel formats.

Source Data Extended Data Fig. 6 Statistical source data—Excel formats.

Source Data Extended Data Fig. 8 Statistical source data—Excel formats

## Data Availability

Source data are provided with this paper. Volumetric data could not be uploaded with the source data due to the large file size and are available from the corresponding author upon request. The crystallographic data for the structures reported here have been deposited at the Cambridge Crystallographic Data Centre (CCDC) under deposition numbers CCDC 2308176 (laser off), 2308180 (time delay −3.9 ps), 2308179 (time delay −2.4 ps), 2308178 (time delay −0.6 ps), 2308184 (time delay −0.2 ps), 2308177 (time delay 0 ps), 2308185 (time delay 0.1 ps), 2308183 (time delay 0.4 ps), 2308181 (time delay 0.6 ps), 2308182 (time delay 0.85 ps), 2308207 (time delay 1.1 ps), 2308208 (time delay 1.35 ps), 2308210 (time delay 1.6 ps), 2308209 (time delay 1.85 ps), 2308205 (time delay 2.1 ps), 2308202 (time delay 2.35 ps), 2308203 (time delay 2.6 ps), 2308201 (time delay 2.85 ps), 2308204 (time delay 3.1 ps), 2308206 (time delay 3.35 ps), 2308220 (time delay 3.6 ps), 2308218 (time delay 4.1 ps), 2308222 (time delay 5.1 ps), 2308221 (time delay 7.1 ps), 2308217 (time delay 10.1 ps), 2308215 (time delay 17.9 ps), 2308214 (time delay 31.7 ps), 2308213 (time delay 56.3 ps), 2308216 (time delay 100.1 ps), 2308219 (time delay 178.1 ps), 2308057 (time delay 316.1 ps), 2308056 (time delay 562.1 ps), 2308055 (time delay 1 ns), 2308058 (time delay 3 ns), 2308737 (I_hot_), 2308738 (I_tr_) and 2308736 (I_osc_). Copies of the data can be obtained free of charge via https://www.ccdc.cam.ac.uk/structures/.

## Extended data

**Extended Data Table 1:** Crystallographic parameters and complete refinement statistics for the extrapolated maps of three structural species

Time delay (ps)	I_osc_	I_tr_	I_hot_	Ground state
**CCDC reference number**	2308736	2308738	2308737	2308176
**Formula**	C_144_ H_72_ Fe_3_ N_12_ O_64_ Zr_12_		C_147_ H_72_ Fe_3_ N_12_ O_67_ Zr_12_	
**Molecular weight**	4,256.32		4,340.35	
**Space group**	$Im\overline{3}m$			
**a, b, c (Å)**	38.873	38.873	38.873	38.873
**α, β, γ (°)**	90	90	90	90
**V (Å** ^**3**^ **)**	58,743	58,743	58,743	58,743
**Z**	4	4	4	4
**ρ**_**calc**_ **(g/cm**^**−3**^**)**	0.481	0.481	0.491	0.491
**λ (Å), T (K)**	0.82656, 293			
**d**_**min**_ **(Å) [I** **>** **2σ(I)]**	0.9	0.9	0.9	0.9
**Observed data [I** **>** **2σ(I)]**	4,116	4,116	4,116	4,118
**Restraints**	221	161	160	143
**Parameters**	127	122	130	131
**R**_**1**_ **(obs) (%)**	13.99	11.88	7.74	13.41
**R**_**1**_ **(all) (%)**	15.04	13.07	9.10	15.96
**Goodness-of-fit on F** ^**2**^	2.08	1.71	1.08	1.32
**Peak/hole (e** **Å**^**−3**^**)**	1.92 −0.72	0.94 −0.73	0.70 −0.24	1.34 −1.05

Z, formula units in unit cell

## Code availability

The code used for the analysis of the different ED maps can be obtained from the corresponding author upon reasonable request.
