# Supplementary material for: Dynamic three-dimensional structures of a metal–organic framework captured with femtosecond serial crystallography
Source: Nat Chem. 2024 Mar 25;16(5):693–9. doi: 10.1038/s41557-024-01460-w (PMC11087265; doi:10.1038/s41557-024-01460-w)
Supplement: Supplementary file 1 — Supplementary Methods, Discussion, Figs. 1–10 and Tables 1–8. [file 41557_2024_1460_MOESM1_ESM.pdf]

nature chemistry

Article

https://doi.org/10.1038/s41557-024-01460-w

Dynamic three-dimensional structures of a metal–organic framework captured with femtosecond serial crystallography

In the format provided by the authors and unedited

Table of Contents

Supplementary Methods

1. Characterization of PCN–224(No metal) and PCN–224(Fe) p.1
2. Kinetic analysis using kinetic modeling p.2

Supplementary Discussion

1. Origin of the absorption of PCN–224(Fe) at 400 nm p.5
2. Data reproducibility and errors in difference electron density maps p.5
3. Comparison of the structural dynamics of PCN–224(Fe) and myoglobin p.7
4. Estimation of the temperature rise in PCN–224(Fe)–CO upon photoexcitation p.7

Supplementary Figures

1. Supplementary Fig. 1 .... p.9
2. Supplementary Fig. 2 .... p.10
3. Supplementary Fig. 3 .... p.11
4. Supplementary Fig. 4 .... p.12
5. Supplementary Fig. 5 .... p.13
6. Supplementary Fig. 6 .... p.14
7. Supplementary Fig. 7 .... p.15
8. Supplementary Fig. 8 .... p.16
9. Supplementary Fig. 9 .... p.17
10. Supplementary Fig. 10 .... p.18

Supplementary Tables

1. Supplementary Table 1 .... p.19
2. Supplementary Table 2 .... p.20
3. Supplementary Table 3 .... p.21
4. Supplementary Table 4 .... p.22
5. Supplementary Table 5 .... p.23
6. Supplementary Table 6 .... p.24
7. Supplementary Table 7 .... p.25
8. Supplementary Table 8 .... p.26

Reference

Supplementary Methods

1. Characterization of PCN–224(No metal) and PCN–224(Fe)

The powder X-ray diffraction (XRD) data were measured on micro-sized crystals (Supplementary Fig. 3). Powder XRD patterns were recorded in the range of 3–145° using a step of 0.01° with a SmartLab diffractometer (Rigaku, Tokyo, Japan) using Cu Kα radiation (λ = 1.5406 Å) at 45 keV and 200 mA. As a reference, the power XRD data of LaB_6 were measured under the same condition, and the data were used to correct the experimental errors (for example, zero-point shift). To determine the lattice parameters of PCN–224(No metal) and PCN–224(Fe) at room temperature, the lattice parameters of two metal–organic frameworks (MOFs) were refined by a whole pattern fit using the Le Bail method implemented in the FULLPROF program[1]. The resulting reflection condition was consistent with the space group ($Im\bar{3}m$) reported in the literature[2]. The observed powder XRD patterns are identical to those previously reported and did not show considerable dependence on the size of crystals. UV-visible spectra were recorded by using a Cary5000 spectrometer (Agilent, Santa Clara, USA) (Supplementary Figs. 4a and 4b). Samples were digested in NaOH solution with pH 12. To confirm the proper incorporation of Fe to PCN–224, diffuse reflectance infrared Fourier transform (DRIFT) spectra were recorded by using a Nicolet iS50 (Thermo Fisher Scientific Instrument, Waltham, USA). The peak at 997.80 cm^{-1} observed in the DRIFT spectrum of PCN–224(Fe) originates from ν(Fe–N), indicating that the Fe metalation was successful (Supplementary Fig. 4c). The inductively coupled plasma atomic emission spectroscopy (ICP-AES) spectra were collected with an ICP-OES 5110 (Agilent, Santa Clara, USA) on samples digested in 70 % aq. HNO_3 : 30 % aq. HCl (7:3 v/v) and heated at 200 °C for 30 min. The ICP-AES spectrum gives the Zr:Fe mass ratio of 4.7 (compared with the expected value of 4.9). Thermogravimetric analysis (TGA) was performed by using a LABSYS evo thermogravimetric analyzer (Setaram, Lyon, France) with the temperature of the activated samples increased from 70 to 600 °C at a rate of 10 °C/min under N_2 (Supplementary Fig. 4d).

2. Kinetic analysis using kinetic modeling

An experimental difference electron density (DED) map at a certain time delay can be considered as a column vector, $\Delta\rho(p)$, where p is an index to denote the position in a three-dimensional lattice, as follows.

$$\Delta\rho(p) = [\,\Delta\rho(p_1),\ \Delta\rho(p_2),\ \cdots\ ,\ \Delta\rho(p_\mathrm{N})\,]^\mathrm{T} \tag{1}$$

The DED map at each time delay is conventionally expressed as a three-dimensional matrix, $\Delta\rho(p_x, p_y, p_z)$. We reduced these three-dimensional matrices with a size of (n_x, n_y, n_z) to a series of column vectors with a size of $(n_x \times n_y \times n_z, 1, 1)$ by reshaping the three-dimensional lattice (p_x, p_y, p_z) into a single dimension of p. Consequently, the time-resolved DED maps, $\Delta\rho(p_x, p_y, p_z, t)$, were also reduced into a two-dimensional matrix, $\Delta\rho(p, t)$, as follows:

$$\Delta\rho(p,t) = [\,\Delta\rho(p,t_1),\ \Delta\rho(p,t_2),\ \cdots\ ,\ \Delta\rho(p,t_\mathrm{M})\,] \tag{2}$$

where each $\Delta\rho(p, t_i)$ is a column vector for a DED map at a certain time delay, t_i. Then, the measured DED map, $\Delta\rho(p, t)$, can be decomposed into two different components, one for the set of time-independent DED maps corresponding to each intermediate species or product and the other for their relative time-dependent contributions to the overall DED maps. Hence,

$$\begin{aligned}&\Delta\rho(p,t)\\ &= [\,\sum_{i=1}^{S}\mathrm{c}_i(t_1)\cdot\mathrm{SADED}_i(p),\ \sum_{i=1}^{S}\mathrm{c}_i(t_1)\cdot\mathrm{SADED}_i(p),\ \cdots\ ,\ \sum_{i=1}^{S}\mathrm{c}_i(t_\mathrm{M})\cdot\mathrm{SADED}_i(p)\,]\\ &= \mathrm{E}(p)\mathrm{C}(t)^\mathrm{T}\end{aligned} \tag{3}$$

where S is the number of reaction species (S = 3 for the specific system studied in this work), $\mathrm{c}_i(t_j)$ denotes the concentration of ith species at t_j time delay, $\mathrm{SADED}_i(p)$ is the DED map corresponding to ith reaction species, i.e., species-associated DED map (SADED) of ith species, $\mathrm{E}(p)$ = [$\mathrm{SADED}_1(p)$, $\mathrm{SADED}_2(p)$, … , $\mathrm{SADED}_S(p)$] and $\mathrm{C}(t)$ = [$c_1(t)$, $c_2(t)$, … , $c_S(t)$]. Here, we theoretically modeled the $\mathrm{C}(t)$ based on the kinetic model described above. The corresponding SADED maps, $\mathrm{E}(p)$, are thus a solution of the following linear algebraic equation:

$$\Delta\rho(p,t) = \mathrm{E}(p)\mathrm{C}(t)^\mathrm{T} \tag{4}$$

The transpose of this equation, $C(t)E(p)^T = \Delta\rho(p, t)^T$, takes a well-known form of AX = B where A is $C(t)$, X is $E(p)^T$, and B is $\Delta\rho(p, t)^T$. Since the number of rows is larger than that of columns in $C(t)$, the equation is over-constrained, whose solution is as follows:

$$X = (A^T A)^{-1} A^T B \tag{5}$$

Thus,

$$\begin{aligned} E(p)^T &= (C(t)^T C(t))^{-1} C(t)^T \Delta\rho(p,t)^T \\ E(p) &= \Delta\rho(p,t)((C(t)^T C(t))^{-1} C(t)^T)^T = \Delta\rho(p,t)(C(t)^+)^T \end{aligned} \tag{6}$$

where $C(t)^+$ is the left pseudoinverse of $C(t)$ defined as follows:

$$C(t)^+ = (C(t)^T C(t))^{-1} C(t)^T \tag{7}$$

In other words, we calculated the left pseudoinverse matrix of $C(t)$ and multiplied its transpose to $\Delta\rho(p, t)$ to obtain $E(p)$. The resulting three SADED maps are shown in Fig. 3A. The calculated DED maps, obtained by calculating $E(p)C(t)^T$ of equation (4) are shown in Supplementary Fig. 5 (high contours) and Supplementary Fig. 6 (low contours), and the residual maps obtained by subtracting the calculated DED maps, $E(p)C(t)^T$, from the experimental DED maps are shown in Supplementary Fig. 7.

Then, we analyzed the kinetics of the three species, I_{osc}, I_{tr}, and I_{hot}. Specifically, we extracted the time profiles of the three species and applied a kinetic analysis on the extracted time profiles. To extract the time profiles, the DED map of each time delay was fitted as a linear combination of the three SADED maps. The time profiles for the I_{hot}, I_{tr}, and I_{osc} were obtained from the coefficients of the linear combination. Finally, the time profiles were fitted as a convolution of an instrument response function (IRF) of ~200 fs full width at half maximum (FWHM) with a sum of exponential rise and decay functions and a damped cosine function. We note that, for the kinetic analysis of the time profiles for I_{tr} and I_{osc}, the time constants obtained from the kinetic analysis of the 2nd RSVs of the Zr_6 node and FeCO site were used without further optimization. In other words, for the fitting of the profiles of I_{tr} and I_{osc}, the time constants for the rise (< 200 fs) and decay (47.1 ± 0.5 ps) of the 2nd RSVs and for the oscillatory motions in the RSVs (a period of 5.55 ± 0.01 ps and a damping constant of 2.68 ± 0.02 ps) were fixed. It can be confirmed that the time profiles of I_{tr} and I_{osc} can be described well with these time constants obtained from the analysis of the 2nd RSVs. For the heating kinetics, the time profile of I_{hot} was fitted as a sum of two exponential

rise functions convoluted with the IRF of ~200 fs FWHM. The two time constants for the exponential rise functions were optimized without a constraint. As a result, the time constants of 1.143 ± 0.005 ps and 11.32 ± 0.07 ps were obtained for the rise of the contribution of thermal motion.

Supplementary Discussion

1. Origin of the absorption of PCN–224(Fe) at 400 nm

The UV-visible absorption spectrum of PCN–224(Fe) exhibits a resemblance to that of Fe-porphyrin, particularly around ~400 nm, indicating that the absorption at 400 nm in PCN–224(Fe) is primarily attributed to the Fe-porphyrin group[2,3]. Furthermore, the similarity in UV-visible absorption spectra between PCN–224(Fe) and Fe-porphyrin implies that the effect of the ordered structure of the chromophores inside the MOF on UV-Vis absorption, specifically the electronic interaction between the chromophores, is not significant. The estimated distance between the Fe-porphyrin groups falls within the range of approximately 10 to 20 Å, which is relatively long. Additionally, as the presence of the Zr_6 node between the Fe-porphyrin groups and the perpendicular geometry of adjacent Fe-porphyrin groups impede π conjugation among the Fe-porphyrin group, interactions among the Fe-porphyrin groups through conjugation are expected to be small. Consequently, these negligible interactions between the Fe-porphyrin groups would have minimal effects on the UV-visible absorption. It is widely known that the absorption of porphyrin at around 400 nm corresponds to the π-π^* transition, localized within the porphyrin orbitals. Thus, it is reasonable to infer that the ordered structure of the MOF and CO binding do not substantially affect the absorption. Taking these findings into account, we conclude that the transient motions observed at the Zr_6 node in the MOF are likely induced by the photon-absorbing Fe-porphyrin sites.

2. Data reproducibility and errors in difference electron density maps

We conducted repeated measurements, referred to as "runs," under the same conditions to enhance the signal-to-noise ratio of the data. Here, a "run" indicates a collection of multiple diffraction patterns at the covered time delays, spanning from negative to positive time delays. To assess data reproducibility, we examined the consistency between the

measured runs. A total of seven runs were collected as summarized in Supplementary Table 8. The data presented in the main text represents the average of all seven runs. Ideally, analyzing each individual run independently to ensure consistent results would be preferable. In reality, however, the number of indexed images in each individual run is insufficient for standalone analysis. Therefore, we categorized the measured data into a total of four subsets and averaged the data corresponding to each subset. The subsets were composed as follows: Subset A: runs 2, 3, 4, 6, and 7; Subset B: runs 1, 2, 4, 5, and 7; Subset C: diffraction images from all runs whose indices are odd-numbered; Subset D: diffraction images from all runs whose indices are even-numbered. To examine the consistency between these four subsets, we generated the DED maps for each subset and performed singular value decomposition (SVD) analysis to compare the resulting right singular vectors (RSVs). The resulting RSVs are presented in Supplementary Fig. 8, where we compared the 1st and 2nd RSVs for all subsets. As discussed in the main text, the 1st RSV primarily describes the kinetics of I_{hot} and the 2nd RSV characterizes the kinetics of I_{osc} and I_{tr}. Remarkably, the comparison reveals a high level of consistency in both the 1st and 2nd RSVs among the subsets. This consistency confirms the reproducibility of all subsets, which supports the overall reproducibility of our data.

In addition to the confirmation of the data reproducibility, the error of a DED map was estimated using the subset DED maps. For that purpose, here we assumed that the standard error of the mean (SEM) of four subset DED maps can represent the error of the DED map shown in our manuscript. We calculated the SEM of four subset DED maps for each time delay to examine the error of our DED maps. To represent the magnitude of resulting errors, isosurface plots were generated for comparison between the DED map and its corresponding error, defined as the SEM calculated from the subset DED maps, at a representative (1 ns) time delay as an example (Supplementary Fig. 9). We note that similar magnitudes of errors were observed for the other time delays as well. The comparison demonstrates that the error values are significantly smaller compared to the DED values, indicating a high degree of reproducibility of the measured data.

3. Comparison of the structural dynamics of PCN–224(Fe) and myoglobin

One of the representative systems containing Fe-porphyrin is myoglobin (Mb), and its structural dynamics have been investigated with TR-SFX. Although PCN–224(Fe) and Mb share similar Fe-porphyrin groups, they exhibit significant differences in their molecular frameworks. For example, an Mb crystal has less void than PCN–224(Fe) and contains densely packed solvent molecules. Furthermore, in Mb, the porphyrin group is surrounded by neighboring residues, forming a tightly packed environment, whereas in PCN–224(Fe), it is encompassed by an empty space. The structures of three structural intermediates unveiled in this work offer an opportunity to study the effect of these differences in molecular frameworks on structural dynamics. Considering the similar chromophores, we compared the structural changes occurring in PCN–224(Fe) and Mb, focusing on the local structural changes around the chromophores. According to a TR-SFX study on Mb, upon photoexcitation, CO dissociation and heme doming occur within their time resolution (~250 fs)[4]. In our study, the structural species I_{tr}, where the CO dissociation and structural changes of Fe-porphyrin take place, emerges within our time resolution of ~200 fs. These results are similar to those observed in Mb. On the other hand, PCN–224(Fe) lacks the progress of the heme doming between 0.6 and 3 ps observed in Mb. Another TR-SFX study proposed the formation of the high spin state in the heme group, characterized by positive DED surrounding iron in a cubic shape, and the occurrence of thermal effects, with negative DED at the iron position surrounded by a shell of positive DED[5]. A DED map corresponding to the high-spin state formation was not observed, but the SADED of I_{hot} exhibits a similar feature due to the thermal effects observed in Mb.

4. Estimation of the temperature rise in PCN–224(Fe)–CO upon photoexcitation

We explored the temperature change in PCN–224(Fe)–CO upon photoexcitation using two different quantitative analysis methods, each offering unique insights.

The first approach involved examining the isotropic B-factor of carbon atoms in the PCN–224 sample. This isotropic B-factor value increases as the temperature rises. A quantitative analysis requires a reference for the temperature dependency of isotropic B-factor specific to the PCN–224 sample, but no such reference exists. Instead, we approximated the temperature dependency of the B-factor in a well-known sample,

specifically, the lysozyme protein[6]. By assuming that the isotropic B-factor values of carbon atoms in PCN–224 exhibit a temperature-dependent behavior similar to that of light atoms (carbon, nitrogen, oxygen, and sulfur atoms) in the lysozyme, we estimated the temperature change experienced by the carbon atoms in PCN–224. In this estimation, we simplified the analysis by assuming the temperature as the only factor affecting the B-factor, while neglecting the potential contribution of directional movements of the atoms. The resulting temperature change in PCN–224 is presented in Supplementary Fig. 10 as a function of the time delay after the photoexcitation. The plot indicates that the temperature of atoms in porphyrin of PCN–224 increased by approximately 35 K at around 1 ns after laser exposure. We term the estimated temperature as Tlight, as this temperature is derived only from the ΔB of light atoms.

In contrast, our second method utilized time-resolved changes of isotropic B-factors (ΔB) of heavy atoms (Fe and Zr). Specifically, the isotropic displacement parameter was calculated from ΔB, with an average value of 0.118 $Å^2$ observed for the heavy atoms (Fe and Zr) over time delays ranging from –4 to –0.3 ps. Subsequently, the temperature scale parameter (k_B) value was derived using a previously described method[7]. The temperature changes were then calculated based on two key assumptions: (1) k_B is directly proportional to temperature, and (2) photo-induced movements, excluding those contributing to the temperature rise, account for approximately 20% of the k_B value. The resulting temperature, denoted as Theavy, deduced from the ΔB of heavy atoms is depicted in Supplementary Fig. 10, where the temperature is estimated to increase by approximately 60 K at 1 ns. This estimate significantly exceeds the rise estimated from the isotropic B-factor of carbon atoms.

The significant difference in the estimated temperature increments derived from the two methods can be attributed to the differing atomic focus. The first method's estimation relies on the behavior of carbon atoms, whereas the second method is focused on heavy atoms. The difference underscores the pivotal role that the choice of atoms plays in these estimations, emphasizing the complexities inherent in precisely determining temperature changes in photoexcited materials.

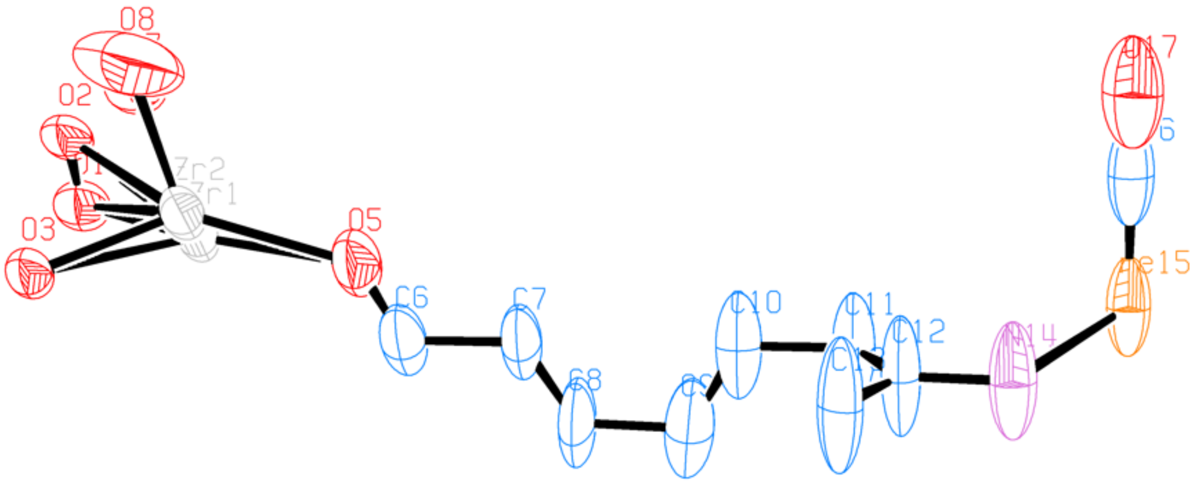

Supplementary Fig. 1. Molecular structure of asymmetric unit of PCN–224(Fe)–CO. Out of 37 CCDC reference numbers, only one (CCDC reference number: 2308176) is shown as an example. Thermal ellipsoids are shown at 30% probability and H atoms were omitted for clarity.

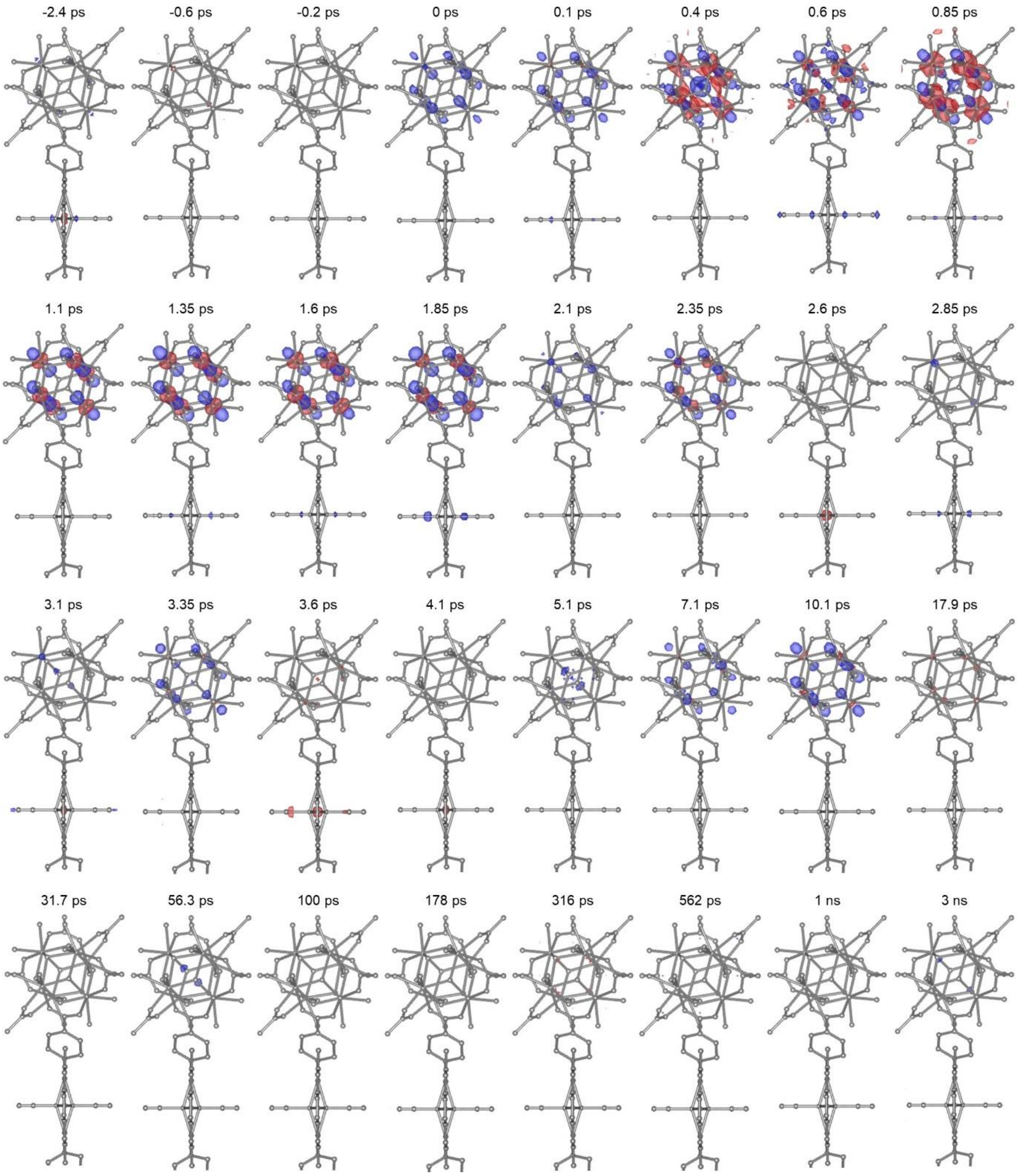

Supplementary Fig. 2. Heat-free time-resolved DED maps of PCN–224(Fe) at all time delays. For each time delay, heat-free DED maps are shown. A heat-free DED map was generated by removing the feature corresponding to the heating component from the DED map of each time delay. The red and blue colors denote the negative and positive electron densities, contoured at ±4.5σ (0.25 e/Å^3).

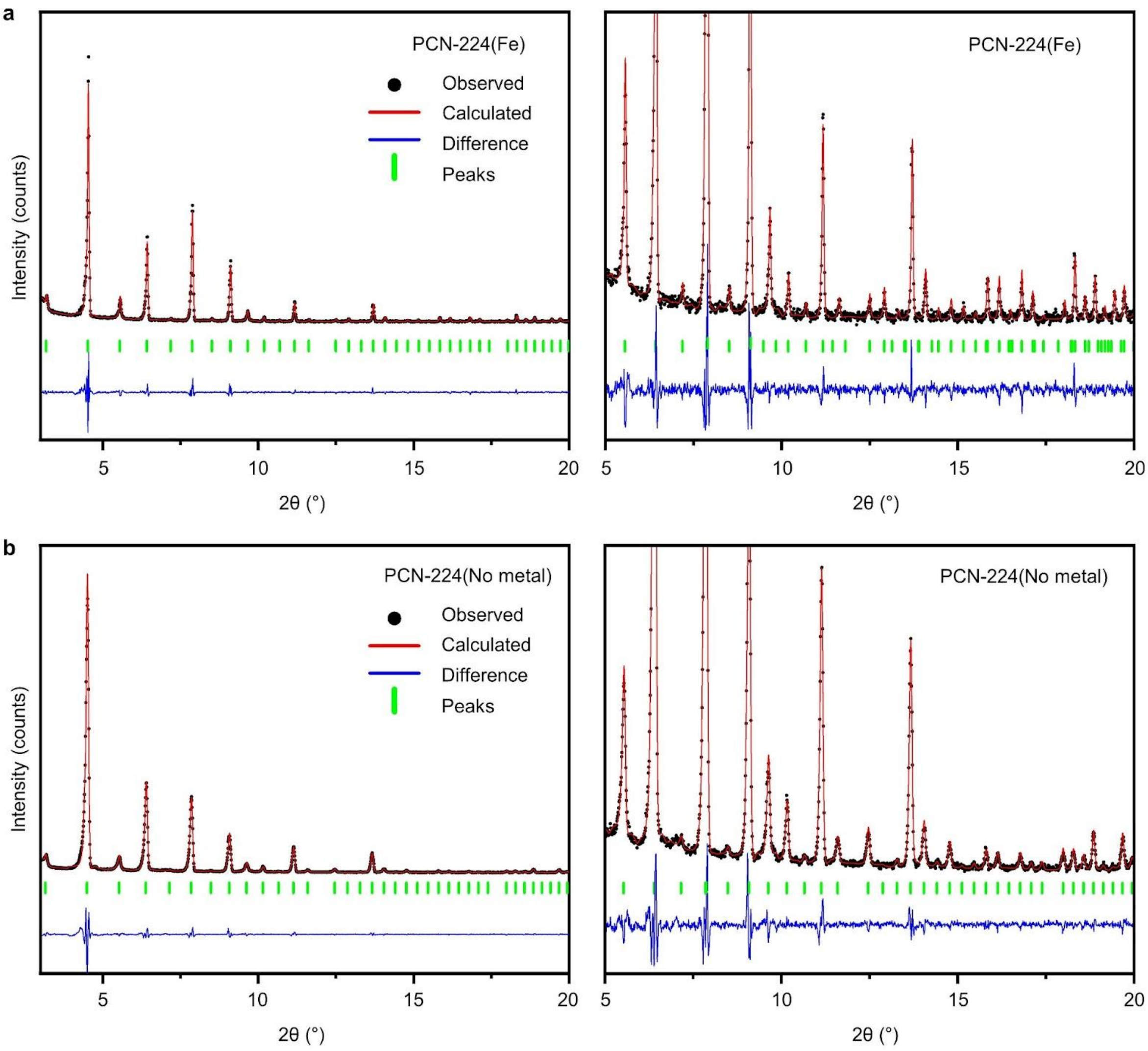

Supplementary Fig. 3. Powder XRD pattern of PCN–224(No metal) and PCN–224(Fe). **a**, Le Bail plot of PCN–224(Fe). The refined unit cell length is a = 38.6097(4) Å. **b**, Le Bail plot of PCN–224(No metal). The refined unit cell length is a = 38.6500(4) Å. The observed powder XRD pattern, calculated pattern, and the difference (observed - calculated) are shown with black circles, red line, and blue line, respectively. The allowed reflections (peaks) are marked with green lines.

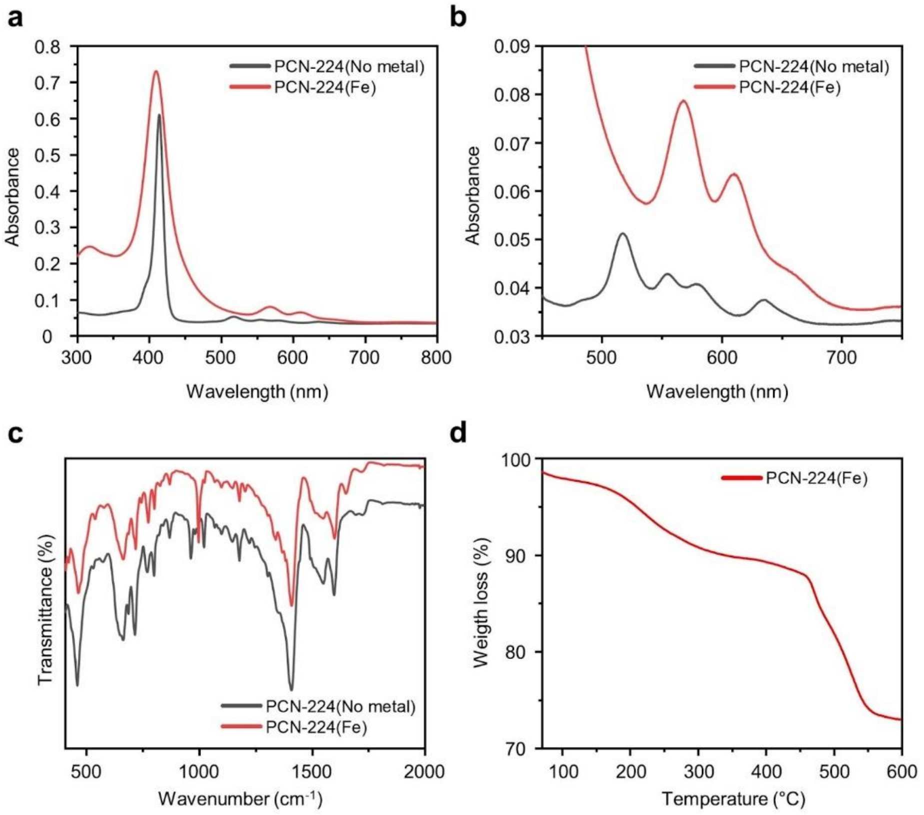

Supplementary Fig. 4. Characterization of PCN–224 samples. a,b, UV-visible absorption spectra of PCN–224(No metal) and PCN–224(Fe) digested in NaOH solution. The spectra measured at 300–800 nm are shown in **a** and their magnified view in the region of 450–750 nm is shown in **b**. For PCN–224(No metal) and PCN–224(Fe), 1 mg of the sample was digested in 50 mL and 10 mL of NaOH solution with pH 12, respectively. **c**, Diffuse reflectance infrared Fourier transform (DRIFT) spectra of PCN–224(No metal) and PCN–224(Fe). The peak at 997.80 cm^{-1} for PCN–224(Fe) originates from ν(Fe–N), indicating that the Fe metalation was successful. **d**, Thermogravimetric analysis trace of PCN–224(Fe). The weight loss around 200–300 °C is due to the removal of the modulators, linkers and dehydroxylation of zirconium nodes. The decomposition temperature is around 472 °C. In all panels, black and red curves are for PCN–224(No metal) and PCN–224(Fe), respectively.

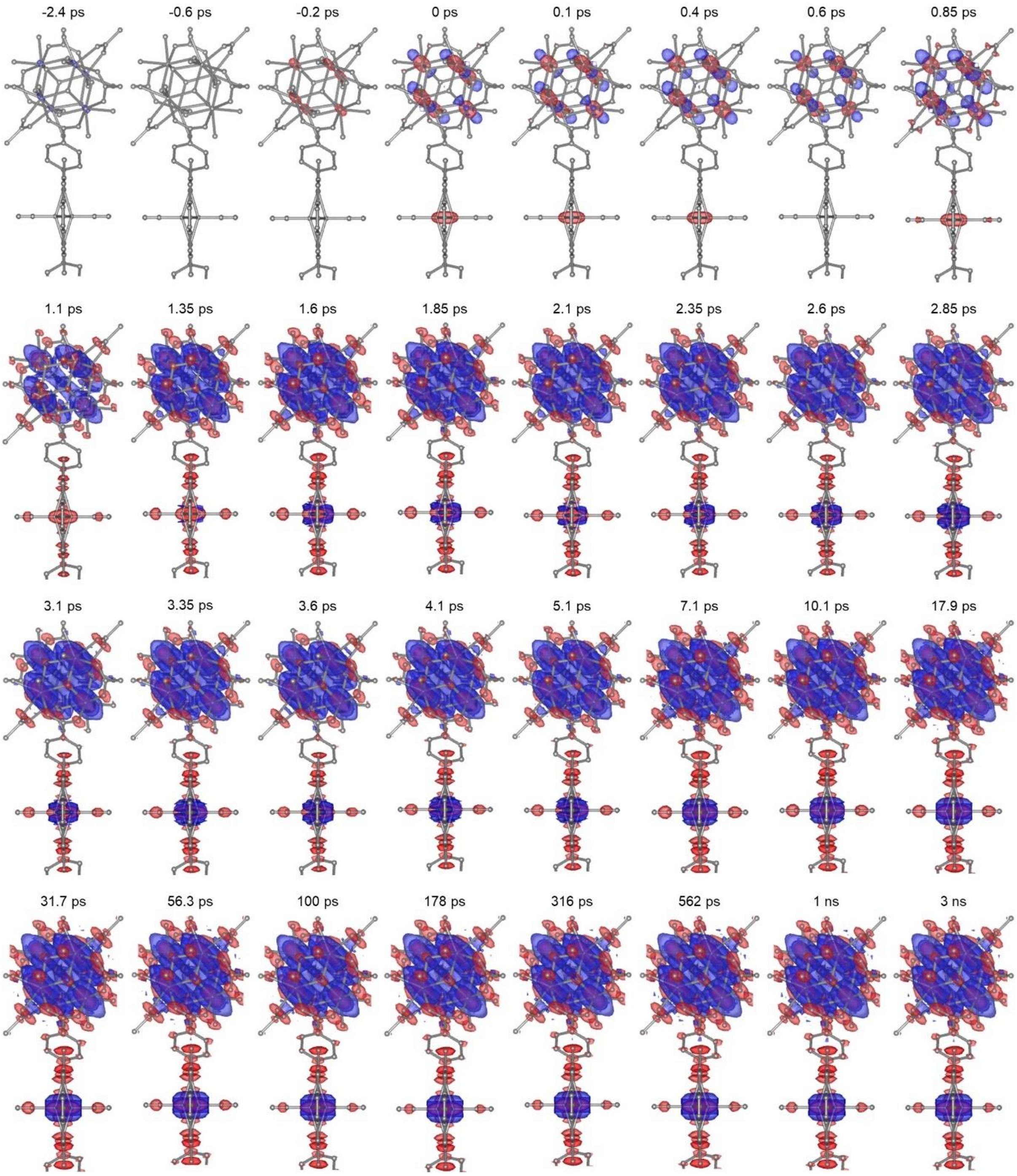

Supplementary Fig. 5. Calculated DED maps of PCN–224(Fe) at all time delays (high contour levels). DED maps including both the Fe-porphyrin and Zr_6 node regions are shown. The red and blue colors denote the negative and positive electron densities, contoured at ±5.0σ (0.5 e/Å^3) respectively.

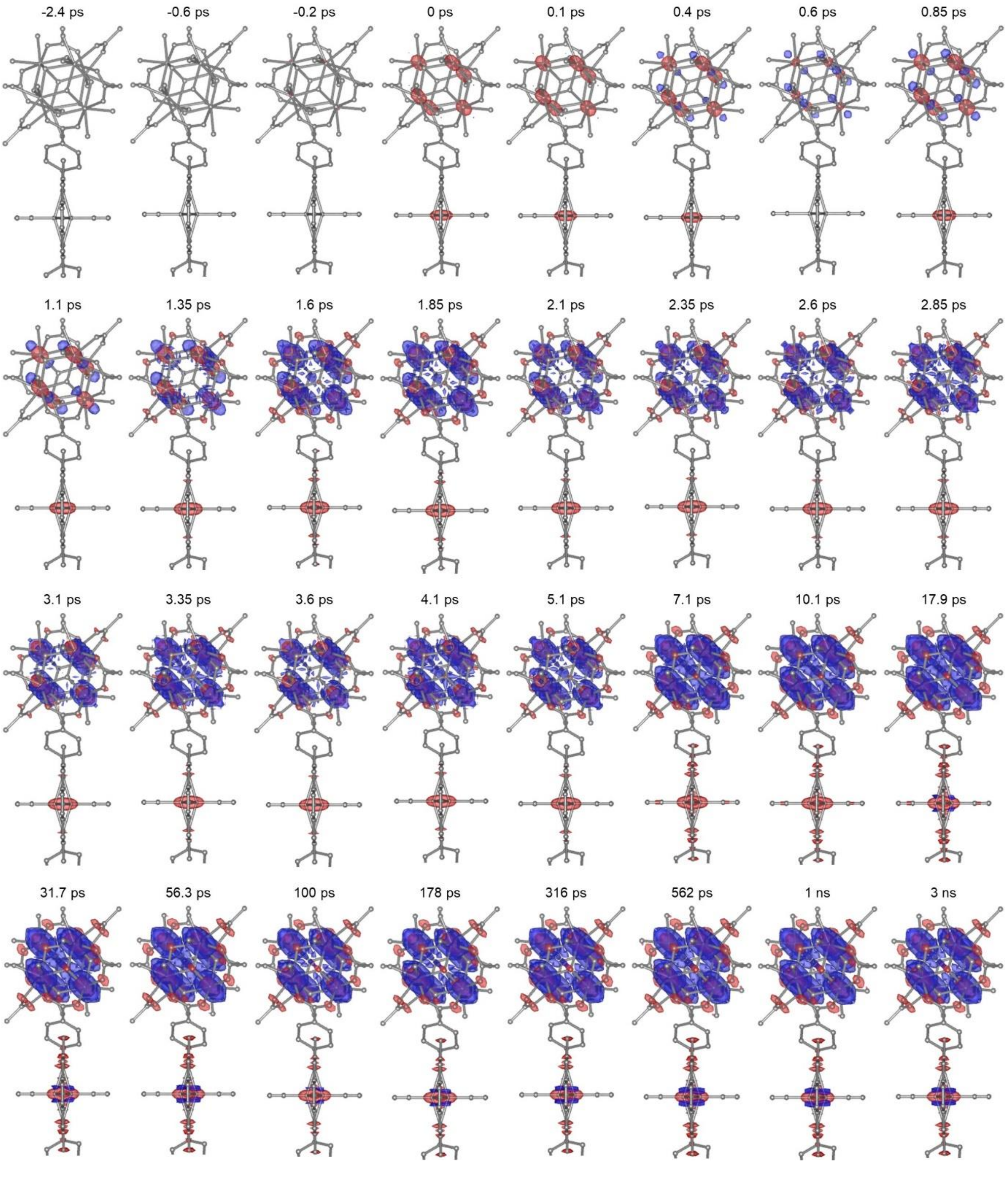

Supplementary Fig. 6. Calculated DED maps of PCN–224(Fe) at all time delays (low contour levels). DED maps including both the Fe-porphyrin and Zr_6 node regions are shown. The red and blue colors denote the negative and positive electron densities, contoured at ±3.0σ (0.3 e/Å^3), respectively.

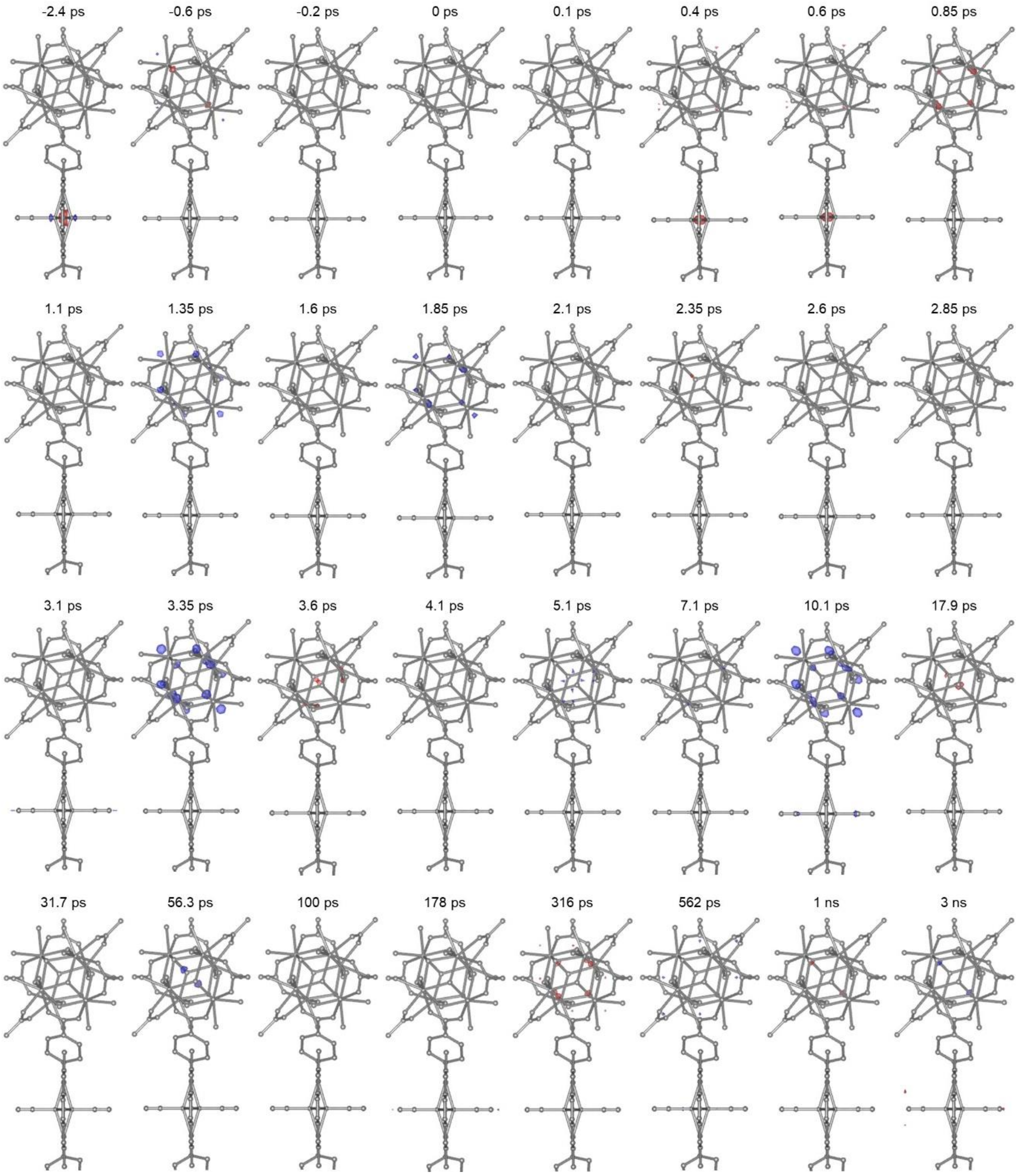

Supplementary Fig. 7. Residual DED maps of PCN–224(Fe) at all time delays (low contour levels). Residual maps, calculated by subtracting the calculated DED maps shown in Supplementary Fig. 5 from the experimental DED maps shown in Extended data Fig. 2, including both the Fe-porphyrin and Zr_6 node regions are shown. The red and blue colors denote the negative and positive electron densities, contoured at ±2.0σ (0.23 e/Å^3), respectively.

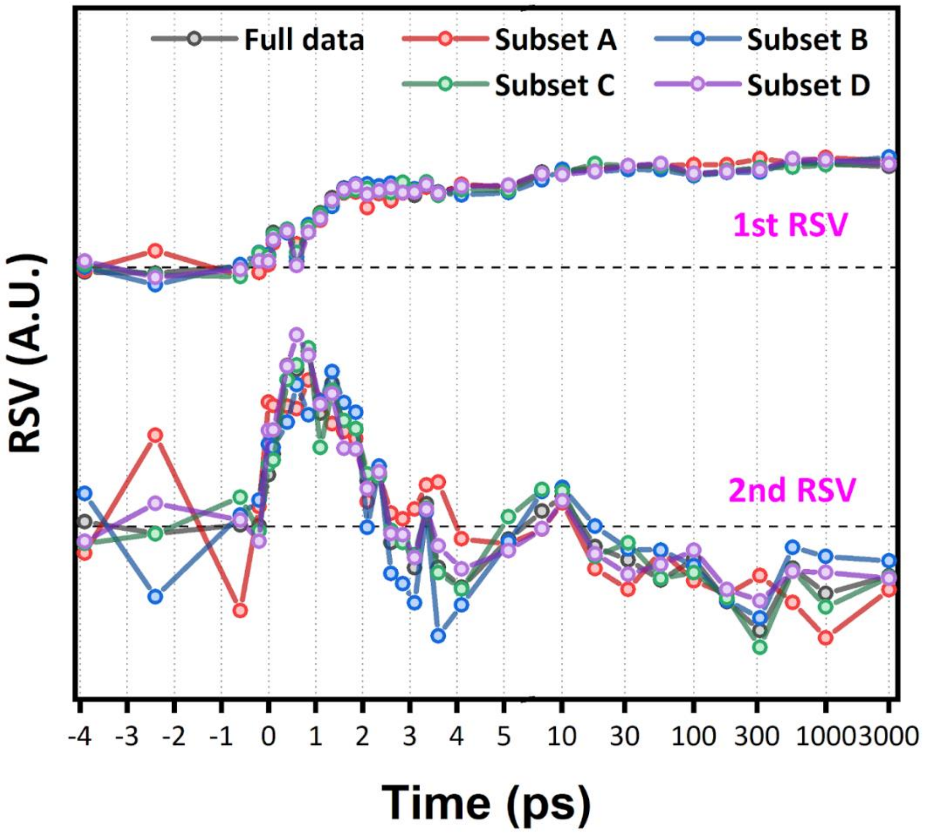

Supplementary Fig. 8. Assessment of data reproducibility. The measured TR-SFX data were partitioned into four overlapping subsets to evaluate the reproducibility and consistency among these subsets. DED maps were generated for each subset of the measured data, and the singular value decomposition (SVD) analysis was applied. The 1st and 2nd right singular vectors (RSVs) for each subset as well as the average of the entire measured dataset were overlaid for comparison. The comparison demonstrates excellent agreement between subsets and the average of the entire data, confirming a high level of data consistency and providing strong support for the reproducibility of the measured data. Further details are provided in the "Data reproducibility and errors in DED maps" section.

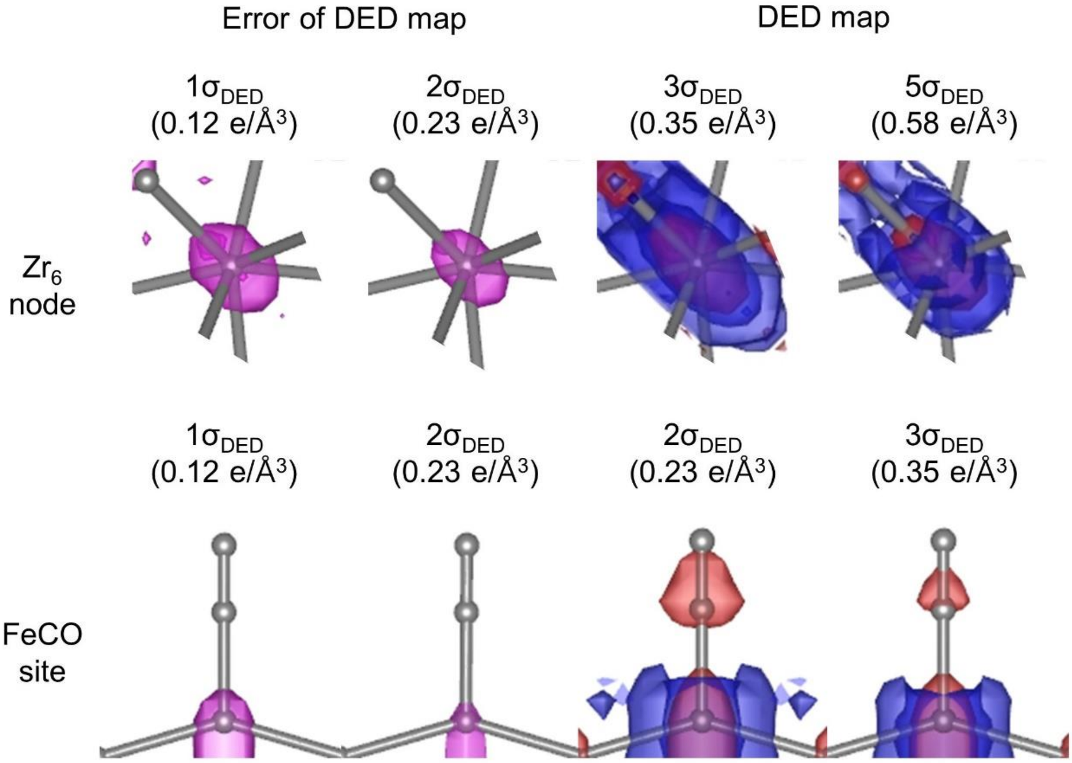

Supplementary Fig. 9. Comparison of the DED values and their respective errors. To assess the reliability of the obtained DED maps, we estimated the errors associated with the DED maps. The error estimation method is provided in the "Data reproducibility and errors in DED maps" section. The resulting error values are depicted in the left panels. The DED maps are displayed in the right panels for comparison. The error and the DED map are displayed for a representative time delay of 1 ns, while similar magnitudes of errors are observed for the other time delays. For the left panels, isosurface maps are shown for two different error values of 0.12 e/Å^3 and 0.23 e/Å^3, corresponding to 1σ and 2σ of the DED map, respectively. In the right DED map panels, two isosurface maps are displayed for 3σ and 5σ (0.35 e/Å^3 and 0.58 e/Å^3) for the region around the Zr_6 node, and 2σ and 3σ for the region around the FeCO site. For the error panels, only the positive isosurfaces corresponding to the absolute magnitude of the error are shown in magenta color. In the DED map panels, positive and negative isosurfaces are represented in blue and red, respectively.

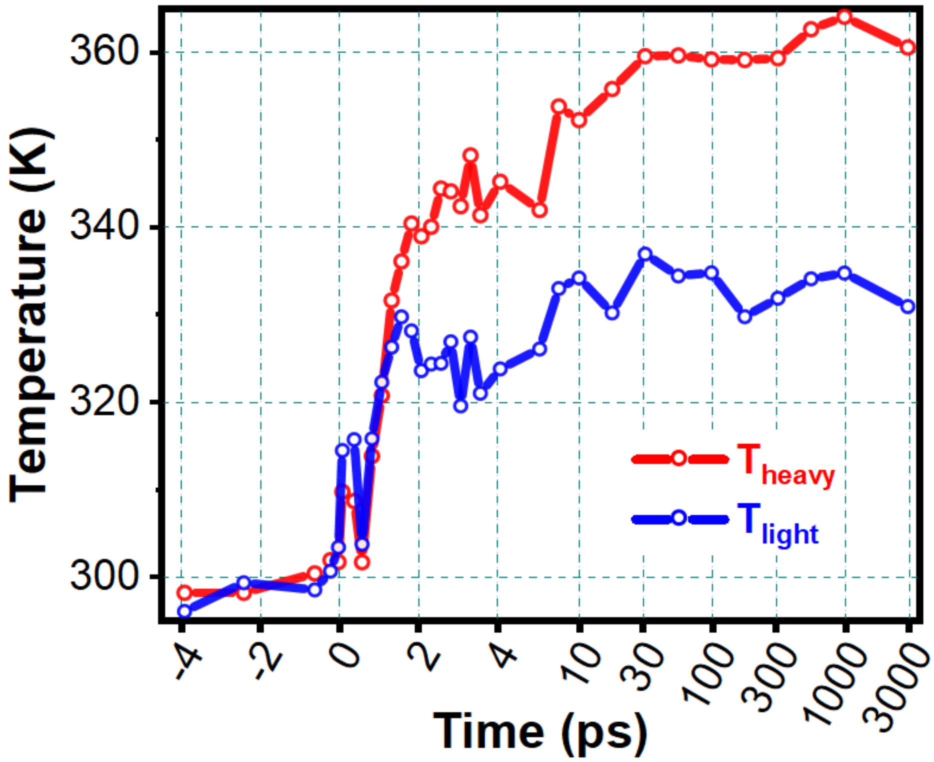

Supplementary Fig. 10. The temporal evolution of temperature (T). The temperatures estimated based on heavy atoms only (T_{heavy}) and light atoms only (T_{light}) are shown in red and blue, respectively. The details are in Supplementary Discussion.

Supplementary Table 1. Crystallographic parameters and complete experimental and refinement statistics for TR-SFX data from PCN–224(Fe)–CO for laser off and time delays from -3.9 ps to 0.1 ps.

Time delay (ps)	Ground state (laser off)	-3.9	-2.4	-0.6	-0.2	0	0.1
CCDC reference number	2308176	2308180	2308179	2308178	2308184	2308177	2308185
Formula	$C_{144} H_{72} Fe_3 N_{12} O_{64} Zr_{12}$						
Molecular weight	4,340.35						
Space group	$Im\bar{3}m$						
a, b, c (Å)	38.873	38.863	38.890	38.877	38.843	38.853	38.903
α, β, γ (°)	90	90	90	90	90	90	90
V (Å^3)	58,743	58,697	58,818	58,758	58,607	58,652	58,879
Z	4	4	4	4	4	4	4
ρ_{calc} (g/cm^{-3})	0.491	0.491	0.49	0.491	0.492	0.492	0.49
λ (Å), T (K)	0.82656, 293						
d_{min} (Å) [I>2σ(I)]	0.9	0.9	0.9	0.9	0.9	0.9	0.9
μ (mm^{-1})	0.448	0.448	0.447	0.448	0.449	0.449	0.447
Frames	50,000	25,000	20,000	20,000	25,000	30,000	30,000
Time (h)	0.46	0.19	0.19	0.19	0.23	0.28	0.28
Crystals	8,825	5,510	3,868	5,145	6,006	7,275	7,944
Data	7,319	7,247	7,309	7,301	7,295	7,319	7,332
Observed data [I>2σ(I)]	4,118	4,116	4,116	4,108	4,104	4,118	4,125
Restraints	143	155	144	143	137	144	143
Parameters	131	131	131	131	131	131	131
R_1 (obs) (%)	13.41	12.91	12.92	13.10	13.08	13.2	13.1
R_1 (all) (%)	15.96	15.40	15.28	15.44	15.45	15.5	15.3
Goodness-of-fit on F^2	1.32	1.27	1.14	1.25	1.28	1.32	1.29
Peak/hole (e^- Å^{-3})	1.34<br>-1.05	1.07<br>-1.00	1.08<br>-0.74	0.98<br>-0.82	1.01<br>-0.85	1.03<br>-0.72	0.94<br>-0.73

Z, formula units in unit cell; ρ, density; μ, absorption coefficient; frames, number of recorded images including both hit and empty ones; crystals, number of indexed images; data, number of unique structure factors in refinement

Supplementary Table 2. Crystallographic parameters and complete experimental and refinement statistics for TR-SFX data from PCN–224(Fe)–CO for time delays from 0.4 ps to 1.85 ps.

**Time delay (ps)**	**0.4**	**0.6**	**0.85**	**1.1**	**1.35**	**1.6**	**1.85**
**CCDC reference number**	2308183	2308181	2308182	2308207	2308208	2308210	2308209
**Formula**	$C_{144} H_{72} Fe_3 N_{12} O_{64} Zr_{12}$						
**Molecular weight**	4,340.35						
**Space group**	$Im\bar{3}m$						
**a, b, c (Å)**	38.883	38.887	38.877	38.883	38.880	38.877	38.880
**α, β, γ (°)**	90	90	90	90	90	90	90
**V (Å^3)**	58,788	58,804	58,758	58,788	58,773	58,758	58,773
**Z**	4	4	4	4	4	4	4
**ρ_{calc} (g/cm^{-3})**	0.49	0.49	0.491	0.49	0.491	0.491	0.491
**λ (Å), T (K)**	0.82656, 293						
**d_{min} (Å) [I>2σ(I)]**	0.9	0.9	0.9	0.9	0.9	0.91	0.91
**μ (mm^{-1})**	0.448	0.447	0.448	0.448	0.448	0.448	0.448
**Frames**	45,000	25,000	50,000	45,000	55,000	45,000	45,000
**Time (h)**	0.42	0.23	0.46	0.42	0.51	0.42	0.42
**Crystals**	9,410	5,208	11,238	9,285	10,342	8,145	8,147
**Data**	7,331	7,321	7,335	7,334	7,313	7,294	7,289
**Observed data [I>2σ(I)]**	4,124	4,119	4,128	4,125	4,114	4,103	4,103
**Restraints**	156	143	158	138	149	150	155
**Parameters**	131	131	140	131	131	131	131
**R_1 (obs) (%)**	12.3	13.2	12.4	12.7	13.1	12.6	12.7
**R_1 (all) (%)**	14.4	15.6	14.5	15.1	15.6	15.4	15.5
**Goodness-of-fit on F^2**	1.35	1.25	1.38	1.30	1.39	1.29	1.29
**Peak/hole (e$^-$ Å^{-3})**	0.90<br>-0.96	1.05<br>-0.89	0.92<br>-1.07	0.88<br>-0.75	0.88<br>-0.74	0.86<br>-0.82	0.87<br>-0.81

Z, formula units in unit cell; ρ, density; μ, absorption coefficient; frames, number of recorded images including both hit and empty ones; crystals, number of indexed images; data, number of unique structure factors in refinement

Supplementary Table 3. Crystallographic parameters and complete experimental and refinement statistics for TR-SFX data from PCN–224(Fe)–CO for time delays from 2.1 ps to 3.6 ps.

Time delay (ps)	2.1	2.35	2.6	2.85	3.1	3.35	3.6
CCDC reference number	2308205	2308202	2308203	2308201	2308204	2308206	2308220
Formula	C_{144} H_{72} Fe_3 N_{12} O_{64} Zr_{12}						
Molecular weight	4,340.35						
Space group	$Im\bar{3}m$						
a, b, c (Å)	38.890	38.88	38.890	38.890	38.883	38.883	38.883
α, β, γ (°)	90	90	90	90	90	90	90
V (Å^3)	58,818	58,773	58,818	58,818	58,788	58,788	58,788
Z	4	4	4	4	4	4	4
ρ_{calc} (g/cm^{-3})	0.49	0.491	0.49	0.49	0.49	0.49	0.49
λ (Å), T (K)	0.82656, 293						
d_{min} (Å) [I>2σ(I)]	0.91	0.91	0.91	0.91	0.91	0.91	0.91
μ (mm^{-1})	0.447	0.448	0.447	0.447	0.448	0.448	0.448
Frames	35,000	40,000	35,000	35,000	35,000	40,000	50,000
Time (h)	0.32	0.37	0.32	0.32	0.32	0.37	0.46
Crystals	6.378	7,146	5,857	5,505	5,103	6,173	7,410
Data	7,278	7,287	7,271	7,256	7,249	7,273	7,283
Observed data [I>2σ(I)]	4,098	4,099	4,094	4,086	4,084	4,095	4,102
Restraints	155	156	156	155	162	156	156
Parameters	131	131	131	131	131	131	131
R_1 (obs) (%)	12.0	12.4	12.2	11.5	11.95	12.3	12.1
R_1 (all) (%)	14.9	15.29	15.4	14.7	15.4	15.8	15.1
Goodness-of-fit on F^2	1.19	1.21	1.20	1.13	1.16	1.21	1.17
Peak/hole (e$^-$ Å^{-3})	0.90<br>-0.80	0.91<br>-0.84	0.77<br>-0.74	0.79<br>-0.81	0.76<br>-0.69	0.86<br>-0.73	0.87<br>-0.77

Z, formula units in unit cell; ρ, density; μ, absorption coefficient; frames, number of recorded images including both hit and empty ones; crystals, number of indexed images; data, number of unique structure factors in refinement

Supplementary Table 4. Crystallographic parameters and complete experimental and refinement statistics for TR-SFX data from PCN–224(Fe)–CO for time delays from 4.1 ps to 56.3 ps.

Time delay (ps)	4.1	5.1	7.1	10.1	17.9	31.7	56.3
CCDC reference number	2308218	2308222	2308221	2308217	2308215	2308214	2308213
Formula	$C_{144} H_{72} Fe_3 N_{12} O_{64} Zr_{12}$						
Molecular weight	4,340.35						
Space group	$Im\bar{3}m$						
a, b, c (Å)	38.880	38.880	38.890	38.877	38.873	38.880	38.883
α, β, γ (°)	90	90	90	90	90	90	90
V (Å^3)	58,773	58,773	58,818	58,758	58,743	58,773	58,788
Z	4	4	4	4	4	4	4
ρ_{calc} (g/cm^{-3})	0.491	0.491	0.49	0.49	0.49	0.491	0.49
λ (Å), T (K)	0.82656, 293						
d_{min} (Å) [I>2σ(I)]	0.91	0.94	0.91	0.91	0.94	0.94	0.91
μ (mm^{-1})	0.448	0.448	0.447	0.448	0.448	0.448	0.448
Frames	55,000	25,000	50,000	40,000	35,000	35,000	35,000
Time (h)	0.51	0.23	0.46	0.37	0.32	0.32	0.32
Crystals	7,805	4,297	8,654	6,481	4,840	4,835	4,794
Data	7,264	7,250	7,284	7,278	7,247	7,248	7,237
Observed data [I>2σ(I)]	4,089	4,079	4,102	4,098	4,080	4,082	4,079
Restraints	156	156	156	162	156	156	150
Parameters	131	131	131	131	131	131	131
R_1 (obs) (%)	12.1	11.7	12.1	11.7	11.4	11.2	11.7
R_1 (all) (%)	15.0	15.4	15.1	15.3	15.1	15.0	15.7
Goodness-of-fit on F^2	1.20	1.11	1.24	1.17	1.08	1.06	1.09
Peak/hole (e$^-$ Å^{-3})	0.82<br>-0.74	0.73<br>-0.77	0.76<br>-0.77	0.80<br>-0.78	0.73<br>-0.76	0.67<br>-0.73	0.73<br>-0.69

Z, formula units in unit cell; ρ, density; μ, absorption coefficient; frames, number of recorded images including both hit and empty ones; crystals, number of indexed images; data, number of unique structure factors in refinement

Supplementary Table 5. Crystallographic parameters and complete experimental and refinement statistics for TR-SFX data from PCN–224(Fe)–CO for time delays from 100 ps to 3,000 ps.

**Time delay (ps)**	**100**	**178**	**316**	**562**	**1,000**	**3,000**
**CCDC reference number**	2308216	2308219	2308057	2308056	2308055	2308058
**Formula**	$C_{144} H_{72} Fe_3 N_{12} O_{64} Zr_{12}$					
**Molecular weight**	4,340.35					
**Space group**	$Im\bar{3}m$					
**a, b, c (Å)**	38.887	38.883	38.887	38.883	38.897	38.89
**α, β, γ (°)**	90	90	90	90	90	90
**V ($Å^3$)**	58,804	58,788	58,804	58,788	58,849	58,818
**Z**	4	4	4	4	4	4
**ρ_{calc} (g/cm^{-3})**	0.49	0.493	0.49	0.49	0.49	0.49
**λ (Å), T (K)**	0.82656, 293					
**d_{min} (Å) [I>2σ(I)]**	0.94	0.91	0.94	0.97	0.96	0.97
**μ (mm^{-1})**	0.447	0.448	0.447	0.448	0.447	0.447
**Frames**	35,000	35,000	35,000	25,000	25,000	25,000
**Time (h)**	0.32	0.32	0.32	0.23	0.23	0.23
**Crystals**	4,975	5,055	4,918	3,790	3,478	3,405
**Data**	7,248	7,240	7,251	7,217	7,206	7,223
**Observed data [I>2σ(I)]**	4,083	4,086	4,086	4,059	4,059	4,114
**Restraints**	156	144	144	138	138	144
**Parameters**	131	131	131	131	131	131
**R_1 (obs) (%)**	11.3	11.05	11.2	11.4	10.9	10.8
**R_1 (all) (%)**	15.0	14.69	14.8	15.6	15.3	14.9
**Goodness-of-fit on F^2**	1.06	1.05	1.04	1.02	1.00	0.97
**Peak/hole ($e^- Å^{-3}$)**	0.80<br>-0.80	0.73<br>-0.70	0.84<br>-0.65	0.65<br>-0.79	0.70<br>-0.67	0.75<br>-0.75

Z, formula units in unit cell; ρ, density; μ, absorption coefficient; frames, number of recorded images including both hit and empty ones; crystals, number of indexed images; data, number of unique structure factors in refinement

Supplementary Table 6. Responses to A/B-level alerts associated with the refined static structure for each time delay.

**THETM01_ALERT_3_B**	**The value of sine(theta_max)/wavelength is less than 0.575**
**CCDC reference numbers for the results related to this warning**	2308176, 2308180, 2308179, 2308178, 2308184, 2308177, 2308185, 2308183, 2308181, 2308182, 2308207, 2308208, 2308210, 2308209, 2308205, 2308202, 2308203, 2308201, 2308204, 2308206, 2308220, 2308218, 2308222, 2308221, 2308217, 2308215, 2308214, 2308213, 2308216, 2308219, 2308057, 2308056, 2308055, 2308058
**Response to alert**	To prevent the intensities of the Bragg spots in the low-resolution region from exceeding the detector's saturation limit, the X-ray flux had to be limited. Consequently, the intensity of the Bragg spots in the high-resolution region was insufficient for detection, resulting in the limited resolution.
**084_ALERT_3_B**	**High wR2 Value (i.e. > 0.25)**
**CCDC reference numbers for the results related to this warning**	2308176, 2308180, 2308179, 2308178, 2308184, 2308177, 2308185, 2308183, 2308181, 2308182, 2308207, 2308208, 2308210, 2308209, 2308205, 2308202, 2308203, 2308204, 2308206, 2308220, 2308218, 2308222, 2308221, 2308215, 2308213
**Response to alert**	In serial femtosecond crystallography, diffraction images are acquired from a large number of different crystals with different sizes and crystallinity. Consequently, errors are introduced while merging data obtained from different crystals. Such errors result in a high wR_2 value for this crystal structure. Moreover, the crystallographic analysis of PCN–224 reveals the presence of MOF–525 structural motifs, which manifest as defects within the crystal matrix. These defects are presumed to be a contributing factor to the elevated wR_2 values noted in the analysis.
**029_ALERT_3_B**	**_diffrn_measured_fraction_theta_full value Low**
**CCDC reference number for the results related to this warning**	2308209
**Response to alert**	Serial femtosecond crystallography employs multiple crystals for data collection. In an ideal scenario with ample experiment time and random crystal orientations, all the crystal orientations can be covered. However, a fraction of crystal orientation, as well as a fraction of the corresponding Bragg spots, may remain unmeasured due to either the limited experiment time or the presence of a preferred orientation of the crystals. Another factor contributing to the unmeasured fraction of Bragg spots is the weak intensities of the Bragg spots in the high-resolution region.

Supplementary Table 7. Responses to A/B-level alerts associated with the structural species (I_{osc}, I_{tr}, and I_{hot}).

**THETM01_ALERT_3_B**	**The value of sine(theta_max)/wavelength is less than 0.575**
**CCDC reference numbers for the results related to this warning**	2308736, 2308738, 2308737
**Response to alert**	To prevent the intensities of the Bragg spots in the low-resolution region from exceeding the detector's saturation limit, the X-ray flux had to be limited. Consequently, the intensity of the Bragg spots in the high-resolution region was insufficient for detection, resulting in the limited resolution.
**084_ALERT_3_B**	**High wR2 Value (i.e. > 0.25)**
**CCDC reference numbers for the results related to this warning**	2308736, 2308738
**Response to alert**	The extrapolated structure factors are derived from the ground-state structure factors, the species-associated difference structure factors and the photoconversion yield. Generally, when a species contributes minorly to the overall data, its associated difference structure factors, extracted from the data, tend to be less reliable compared to those with a higher contribution. Consequently, the structure factors of Iosc and Itr, which have relatively minor contributions to the overall data, are extracted with reduced accuracy. This results in the extrapolated structure factors for these two species, I_{osc} and I_{tr}, exhibiting comparatively higher values. In contrast, for I_{hot}, which makes a dominant contribution to the overall data, the species-associated difference structure factors can be extracted with enhanced accuracy. This higher level of accuracy leads to a smaller wR_2 value for Ihot.

Supplementary Table 8. Summary of statistics for each of the seven measured runs. We repeated measurements seven times under the same experimental conditions and for the same or highly similar time delay points, and the data presented in the main text is the average of these measurements (runs) This table provides information for each run, including the time delay range, the number of time delays, the number of measured diffraction images per time delay, and the mean number of indexed images per time delay.

	Time delay range (ps)	Number of time delays	Number of measured diffraction images per time delay	Mean number of indexed images per time delay
Run 1	-3.9 ~ 316	27	10,000	1,750
Run 2	-3.9 ~ 3,000	30	5,000	736
Run 3	-3.9 ~ 3,000	30	5,000	927
Run 4	-3.9 ~ 5.1	20	5,000	497
Run 5	-3.9 ~ 3,000	30	5,000	764
Run 6	-3.9 ~ 3,000	30	5,000	756
Run 7	-3.9 ~ 3,000	43	5,000	1,306

Reference

1. Rodríguez-Carvajal, J. Recent advances in magnetic structure determination by neutron powder diffraction. *Physica B* **192**, 55-69 (1993).
2. Anderson, J. S., Gallagher, A. T., Mason, J. A. & Harris, T. D. A Five-Coordinate Heme Dioxygen Adduct Isolated within a Metal–Organic Framework. *J. Am. Chem. Soc.* **136**, 16489-16492 (2014).
3. Kucheryavy, P., Lahanas, N. & Lockard, J. V. Spectroscopic Evidence of Pore Geometry Effect on Axial Coordination of Guest Molecules in Metalloporphyrin-Based Metal Organic Frameworks. *Inorg. Chem.* **57**, 3339-3347 (2018).
4. Barends, T. R. M. *et al.* Direct observation of ultrafast collective motions in CO myoglobin upon ligand dissociation. *Science* **350**, 445-450 (2015).
5. Ren, Z. Ultrafast Structural Changes Decomposed from Serial Crystallographic Data. *J. Phys. Chem. Lett.* **10**, 7148-7163 (2019).
6. de Sá Ribeiro, F. & Lima, L. M. T. R. Linking B-factor and temperature-induced conformational transition. *Biophys. Chem.* **298**, 107027 (2023).
7. Schmokel, M. S., Kaminski, R., Benedict, J. B. & Coppens, P. Data scaling and temperature calibration in time-resolved photocrystallographic experiments. *Acta Cryst. A* **66**, 632-636 (2010).
